# Ndufa4 Regulates the Proliferation and Apoptosis of Neurons via miR-145a-5p/Homer1/Ccnd2

**DOI:** 10.1007/s12035-023-03239-5

**Published:** 2023-02-10

**Authors:** Fang Fu, Chen Chen, Kun Du, Lu-shan Li, Ru Li, Ting-ying Lei, Qiong Deng, Dan Wang, Qiu-xia Yu, Xin Yang, Jin Han, Min Pan, Li Zhen, Li-na Zhang, Jian Li, Fa-tao Li, Yong-ling Zhang, Xiang-yi Jing, Fu-cheng Li, Dong-zhi Li, Can Liao

**Affiliations:** 1 Department of Prenatal Diagnostic Centre, Guangzhou Women and Children’s Medical Center, Guangzhou Medical University, No.9 of Jinsui Road of Guangzhou, Guangzhou, 510623 Guangdong China; 2 Department of Respirator, Guangzhou Women and Children’s Medical Center, Guangzhou Medical University, Guangzhou, 510623 China

**Keywords:** Ndufa4, Neuron, Proliferation, Apoptosis, miR-145a-5p, *Homer1*

## Abstract

The Dandy–Walker malformation (DWM) is characterized by neuron dysregulation in embryonic development; however, the regulatory mechanisms associated with it are unclear. This study aimed to investigate the role of NADH dehydrogenase 1 alpha subcomplex 4 (NDUFA4) in regulating downstream signaling cascades and neuronal proliferation and apoptosis. *Ndufa4* overexpression promoted the proliferation of neurons and inhibited their apoptosis in vitro, which underwent reverse regulation by the *Ndufa4* short hairpin RNAs. *Ndufa4*-knockout (KO) mice showed abnormal histological alterations in the brain tissue, in addition to impaired spatial learning capacity and exploratory activity. *Ndufa4* depletion altered the microRNA expressional profiles of the cerebellum: *Ndufa4* inhibited miR-145a-5p expression both in the cerebellum and neurons. miR-145a-5p inhibited the proliferation of neurons and promoted their apoptosis. *Ndufa4* promoted and miR-145a-5p inhibited the expression of human homer protein homolog 1 and cyclin D2 in neurons. Thus, *Ndufa4* promotes the proliferation of neurons and inhibits their apoptosis by inhibiting miR-145a-5p, which directly targets and inhibits the untranslated regions of *Homer1* and *Ccnd2* expression.

**Supplementary Information:**

The online version contains supplementary material available at 10.1007/s12035-023-03239-5.

## Introduction

The Dandy–Walker malformation (DWM), or Dandy–Walker syndrome, is a severe congenital posterior fossa anomaly characterized by vermis agenesis and hypoplasia, cystic enlargement of the fourth ventricle, meningeal anomalies, occipital skull defects, and hydrocephalus [1–3]. The incidence rate of DWM is between 1/200 and 1/35000 in different regions, with a mortality rate of > 12%, accounting for ~ 4% of hydrocephalus cases in the USA [1, 2, 4]. DWM originates during embryonic development of the cerebellum and fourth ventricle, and patients commonly present with non-neurologic comorbidities such as mental health and learning disability, endocrine and metabolic diseases, eye and ear disorders, circulatory system disorders, and even neoplasms [5]. Clinical treatment available at present mainly involves surgery, including ventriculoperitoneal shunting, cystoperitoneal shunting, and endoscopic third ventriculostomy, to alleviate hydrocephalus, posterior fossa symptoms, and other associated comorbidities [1, 6]. DWM is a sporadic disorder and can be caused by atresia of the Luschka and Magendie foramina and developmental abnormalities of the rhombencephalon [1, 7]. These result in vermian development arrest and fourth ventricle foramina fenestration failure, which is also associated with chromosomal abnormalities, congenital infections, and environmental exposure [1, 7]. However, the molecular mechanisms underlying DWM development are still unclear.

NADH dehydrogenase (ubiquinone) 1 alpha subcomplex 4 (NDUFA4) is a subunit of complex I in the mitochondrial respiratory chain, which is involved in the assembly and functioning of cytochrome c oxidase (COX, complex IV) during mitochondrial electron transport chain and aerobic metabolism [8–10]. NDUFA4 plays an essential role in mitochondrial function and energy metabolism, and *NDUFA4* expression and mutations are involved in the development of various cellular processes and human disorders such as gastric cancer, clear-cell renal cell carcinoma, colorectal cancer, and diabetes mellitus [11–14]. For instance, the overexpression of *NDUFA4* promotes the proliferation of gastric cancer cells and inhibits their apoptosis, which mediates long noncoding RNA macrophage migration inhibitory factor antisense RNA1-regulated pathogenesis of gastric cancer [12]. In addition, *NDUFA4* expression promotes the proliferation, migration, invasion, and apoptosis of colorectal cancer cells [11]. NDUFA4 is also characterized as one interacting protein of DJ1 (Parkinson disease protein 7) linked with epigenetic regulation and apoptosis pathways in multiple sclerosis development [15]. Therefore, *NDUFA4* is a critical regulator of cell proliferation and apoptosis associated with the pathogenesis of many human disorders.

NDUFA4 also has a crucial role in neuronal functions and the development of neurological diseases. Genetic association analysis of > 1500 clinical samples showed that *NDUFA4* mutation is closely associated with the development of Alzheimer’s disease (AD) [16]. Quantitative proteomic analysis of mitochondrial proteomes showed that NDUFA4 levels in the brain tissue of patients with AD were substantially altered compared with those in healthy individuals [17]. In addition, homozygous splice donor site mutations are characterized in patients with Leigh syndrome by neurological phenotypes such as dystonia, ataxia, bulbar dysfunction, and intermittent encephalopathy [18]. In our previous studies, array-based comparative genomic hybridization (array-CGH) based on three DWM fetuses showed that the chromosome 7p21.3 region containing *NDUFA4* was critically associated with DWM [19]. Additionally, DWM fetuses frequently have *NDUFA4* haploinsufficiency and copy number variations (CNVs) [20]. NDUFA4 was found to effectively enhance the growth and inhibit the apoptosis of neurons by promoting B-cell lymphoma-2 (BCL-2) expression and inhibiting caspase-3 cleavage and cytochrome c (Cyt C) expression and release [21]. Moreover, NDUFA4 expression was found to considerably modulate the regulatory effects of cyclosporin A on neuron growth and apoptosis [21]. All the above studies confirmed that NDUFA4 expression may participate in DWM pathogenesis. However, how NDUFA4 affects neuron growth and apoptosis in embryonic development remains unclear.

Epigenetic regulations mediated by microRNAs have an essential role in various cellular biological processes and pathogenic conditions [22, 23]. However, little is known about the role of *Ndufa4*-regulated microRNAs. This study investigated the potential functions of *Ndufa4*-regulated microRNAs in neuronal proliferation and apoptosis, in addition to downstream target genes and signaling cascades, using a cellular neuronal differentiation model, which was established by treating pluripotent mouse embryonal carcinoma cells (P19 cell line) with all‐trans‐retinoid acid (RA). Our data would reveal novel clues regarding neuron growth and apoptosis in embryonic development.

## Materials and Methods

### Cell Culture and Modeling

We purchased P19 cells from the American Type Culture Collection. Cells were cultivated with Minimal Essential Medium—alpha modification (αMEM #12,571,048; Thermo Fishier Scientific, Waltham, MA, USA) containing 2 mM L-glutamine, 10% fetal bovine serum (Gibco), 100 units/mL of penicillin, and 100 μg/μL of streptomycin in a humidified 5% CO_2_ atmosphere at 37 °C. Cell authentication was done using a short tandem repeat DNA profiling assay. Neural differentiation of cultured P19 cells was induced, as previously described [24]. Briefly, we cultured P19 cells under normal conditions in αMEM supplemented with 1 μM RA (#R4643; Sigma-Aldrich, St. Louis, MO, USA) for 4 d at 37 °C.

### Cell Transfection

Short hairpin RNA (shRNA) targeting *Ndufa4* (shRNA: 5′-GGAACAAACTGGGTCCCAATG-3′) was obtained from GenePharma (Shanghai, China) and integrated into the pSicoR-Ef1a-mCh-Puro vector (Addgene, Watertown, MA, USA). LV003 vectors for *Ndufa4* overexpression were obtained from General Biology (Anhui, China). The 3′ untranslated regions (3′UTRs) of *Ndufa4*, human homer protein homolog 1 (*Homer1*), and cyclin D2 (*Ccnd2*) were also obtained from General Biology and ligated with the pmirGLO vector. The miR-145a-5p inhibitor (5′-GUCCAGUUUUCCCAGGAAUCCCU-3′), its negative control (5′-UCCAUCAAUCCCGUUCGUGCAGU-3′), miR-145a-5p mimic (5′-GUCCAGUUUUCCCAGGAAUCCCU/GGAUUCCUGGGAAAACUGGACUU-3′), and its negative control (5′-UGAUCUAGUGCCCGAACCCUCUU/AAGAGGGUUCGGGCACUAGAUCA-3′) were obtained from GenePharma. Small interfering RNAs (siRNA) targeting *Ndufa4l2* (sense 5′-CCCGCUUCUACCGGCAGAUTT-3′ and antisense 5′-GGAACCGCAUGAGUCCCAATT-3′) and its negative control (sense 5′-UUCUCCGAACGUGUCACGUTT-3′ and antisense ACGUGACACGUUCGGAGAATT) was obtained from GenePharma. All recombinant vectors and sequences were introduced into cultured P19-derived neurons with Lipofectamine 2000 Reagent (Thermo Fisher Scientific) according to the manufacturer’s instructions.

### Cell Proliferation

After treatment, the proliferation of P19-derived neurons was evaluated by 3-(4,5-dimethylthiazol-2-yl)-5-(3-carboxymethoxyphenyl)-2-(4-sulfophenyl)-2H-tetrazolium (MTS) assay using the Cell Proliferation Colorimetric Assay Kit (#K300-250; BioVision, Milpitas, CA, USA) according to the manufacturer’s instructions. Briefly, P19-derived neurons were collected by centrifugation at 500 × *g* for 5 min, washed twice with phosphate-buffered saline (PBS), and then seeded on 96-well plates. They were incubated with the MTS reagent (25 µL/well) for another 1.5 h at 37 °C, after which their optical density was measured at 490 nm (OD490) using a multiplex plate reader. The OD490 values from at least three biological replicates were measured for comparing the P19-derived neuronal proliferation rates.

### Cell Apoptosis

The P19-derived neurons were processed using the Annexin V-APC/7-aminoactinomycin D (7-AAD) Apoptosis Kit (#KA3808; Abnova, Taipei, Taiwan) according to the manufacturer’s instructions. Briefly, ~ 1 × 10^5^ P19-derived neurons were harvested by centrifugation, washed with PBS, resuspended in 100 µL of binding buffer, and incubated with gentle vortexing in 5 μL of Annexin V-APC and 5 μL of 7-AAD solution for 30 min at room temperature in the dark. Finally, the P19-derived neurons were washed again with PBS and the percentage of apoptotic P19-derived neurons was computed by flow cytometry.

### Ndufa4-Knockout (KO) Mice

The whole body knockout of Ndufa4 had its limitations, but conditional knockout mice were difficult to obtain due to its long construction cycle, so our experiment was still conducted with the whole body knockout mice.

The *Ndufa4*-KO mouse model established by the Cre/LoxP system, as previously described, was purchased from Cyagen Biosciences (Guangzhou, China). The methods of generating *Ndufa4*-KO mice are shown as follows or in Supplemental Figure S1.

The mouse *Ndufa4* gene (NM_010886.3) contains four exons. Exon 3 and 4 were selected as the constitutive KO region. Homologous arms containing upstream and downstream sequences of exon 3 and 4 were amplified by polymerase chain reaction (PCR) using the template DNA extracted from a BAC clone (4E12) to engineer the targeting vector. Then, the homologous arms were sequentially assembled to the 5' and 3' of a loxP-flanking PGK-neo cassette for positive selection. A diphtheria toxin A cassette for negative selection was located upstream of the 5' homologous arm. The targeting vector linearized with NotI was electroporated into C57BL/6 ES cells, followed by G418 antibiotic selection, PCR, and Southern blot validation. After correctly confirming targeted ES clones via Southern blotting, two clones were selected for blastocyst microinjection to produce the F0 generation. The F0 was bred with EIIa-cre mice from the Jackson Laboratory (strain #: 003,724) to delete the PGK-neo cassette. Homozygous F2 was acquired by mating the F1 heterozygotes. The mice were validated using PCR with the primers listed below.

- Ndufa4_F1 (5′-3′): TCATCTCAATCTCGCCTCCCCA.
- Ndufa4_R1 (5′-3′): GAGAGAAGCTGGAAGCAGTCG.
- Ndufa4_R2 (WT) (5′-3′): CACAGAACACCACTCTTTGGGAT.

All mice were kept in a specific pathogen-free-grade atmosphere in a 12/12 h day/night cycle at 20 °C–26 °C. They were fed a standard diet after sterilization, with free access to drinking water. Finally, the mice were euthanized using intraperitoneal 4% chloral hydrate, and mouse brain tissue was collected surgically.

All experimental procedures using mice were approved by the Experimental Animal Care and Ethics Committee of the Forevergen Medical Laboratory Animal Center, Guangzhou, China (Approval no: IACUC-G16051).

### Mouse Behavior Evaluation

The Morris water maze and open-field tests were used to assess the effects of *Ndufa4* KO on mouse behaviors, as previously described [25, 26]. The Morris water maze test analyzed the spatial learning capacity of *Ndufa4*-KO mice. Briefly, the mice were placed at one of four starting spots in a pool and their latency time (s), path length (mm), times on the platform, and time in target quadrants (s) were recorded using the EthoVision system version 2.3 (Noldus, the Netherlands). Next, after dark-adapting the mice for 25 min for the open-field test, they were placed in a 50 × 50 cm open-field arena. To evaluate their exploratory activities, the total distance traveled (mm), number of crossings, center distance (mm), and center time (s) was recorded using the EthoVision system.

### Hematoxylin and Eosin (H&E) Staining

The histological alterations in the brain tissue were analyzed using H&E staining with a commercialized H&E staining kit (#ab245880; Abcam, Cambridge, UK) according to the manufacturer’s instructions. Briefly, the brain sections were deparaffinized, hydrated in distilled water (DW), and incubated in Mayer’s hematoxylin for 6 min at room temperature. Next, they were rinsed twice with DW, incubated in bluing reagent for 15 s at room temperature, and incubated again in Eosin Y solution (Modified Alcoholic; Abcam) for 3 min. Finally, these sections were rinsed and dehydrated with absolute alcohol and then cleared and mounted with synthetic resin.

### Terminal Deoxynucleotidyl Transferase-Mediated dUTP-Biotin Nick End Labeling Assay (TUNEL) Staining

The apoptosis in brain tissue was analyzed using TUNEL staining with a commercialized TUNEL staining kit (#C1086; Beyotime Biotechnology, Shanghai, China) following the manufacturer’s instructions. Briefly, the sections of brain tissue were deparaffinized and hydrated, then incubated with protease K (20 µg/ml) for 20 min. Next, these sections were rinsed thrice with PBS and incubated in the TUNEL solution for 60 min, avoiding light at 37 °C. Finally, the sections were rinsed thrice with PBS, followed by mounting with an antifade mounting medium (#P0128S; Beyotime Biotechnology). The signals were captured using a fluorescence microscope.

### Transmission Electron Microscopy (TEM)

The subcellular structures of brain tissue were observed using TEM. Briefly, the fresh brain tissue was fixed in TEM fixative solution for 2 h at 4 °C, washed thrice with 0.1 M PBS for 15 min, and dehydrated for 15 min using a graded series of ethanol solution (50–100%), followed by 100% acetone for 15 min. Subsequently, the brain tissue was embedded in Spurr’s EPON 812 Resin (#02,660-AB; Emicron, Egypt) by heating it at 60 °C for 48 h and then sliced into 60-nm-thick sections. Finally, these sections were stained with 2% alcohol-saturated uranium acetate solution for 15 min, incubated in lead citrate for 15 min, dried, and observed using TEM.

### Transcriptome Profiling and Bioinformatics

Differentially expressed microRNAs and messenger RNA (mRNA) profiles were detected in mouse brain tissues caused by *Ndufa4* KO using next-generation deep sequencing. Briefly, the total RNA samples from the brain tissue were isolated using the MagMAXmirVana Total RNA Isolation Kit (#A27828; Thermo Fishier Scientific) according to the manufacturer’s instructions. Next, the samples were analyzed using a NanoDrop 2000 spectrophotometer (Thermo Fishier Scientific) to evaluate the RNA quality and concentration, separated using polyacrylamide gel electrophoresis (PAGE), and the isolated RNA bands were arranged in 18–30 nt using the Small RNA PAGE extraction kit (KA4434; Abnova) according to the manufacturer’s instructions. Subsequently, ligation was performed with 3′- and 5′-adaptors and reverse transcription (RT)–PCR to construct a sequencing complementary DNA (cDNA) library. The quality of the cDNA library was assessed using the Agilent 2100 Bioanalyzer (Agilent Technologies, Santa Clara, CA, USA), which was then denatured to single-stranded DNA (ssDNA) and sequenced using the Illumina NextSeq 500 platform (Illumina, San Diego, CA, USA) for 52 cycles. Next, the reads that were obtained were filtered using SolexaSolexa CHASTITY to select clean reads, which were used for subsequent adaptor trimming and alignment with the miRbase database. Tag counts were applied to evaluate the expressional levels of microRNA or mRNA. Significantly different microRNA and mRNA expression was defined as a fold-change (FC) of > 1.5 and *P* < 0.05. The microRNA target genes and their interaction networks were predicted using Targetscan software release 3.1 (www.targetscan.org/mamm_31/) [27]. Lastly, hierarchical clustering of differentially expressed microRNAs or mRNAs was completed using the R software.

### Real-Time Quantitative PCR (qPCR) and Droplet Digital PCR

The relative mRNA or microRNA levels were determined using real-time qPCR. Briefly, total RNA samples were extracted from cultured P19-derived neurons or brain tissue using TRIzol reagent (#15,596,026; Thermo Fishier Scientific) according to the manufacturer’s instructions. NanoDrop 2000 (Thermo Fishier Scientific) was used to measure RNA concentrations. Next, cDNA samples were prepared using 2 µg of RNA from each group by RT using M-MLV Reverse Transcriptase (#M1701; Promega Corporation, Madison, WI, USA) according to the manufacturer’s instructions. Real time-qPCR assay was performed using 2 × SYBR Green PCR Mastermix (#SR1110; SolarBio Life Sciences, Beijing, China) according to the manufacturer’s instructions. At least three biological replicates were performed for relative expression quantitation, with glyceraldehyde 3-phosphate dehydrogenase (GAPDH) or ribosomal protein L7 (RPL7) as the internal standard for mRNA and U6 as the internal standard for microRNA. The sequences of primers were as follows: Ndufa4-F: GTATGTGATGCGCTTGGCAC; Ndufa4-R: TGTTCCATGGCTCTGGGTTG; GAPDH-F: AGGTCGGTGTGAACGGATTTG; GAPDH-R: TGTAGACCATGTAGTTGAGGTCA; RPL7-F: TTGATTGCTCGGTCTCTTGGTAA; RPL7-R: CTGGTCTTCCCTGTTGCCAG; mmu-miR-205-5p-RT: GTCGTATCCAGTGCAGGGTCCGAGGTATTCGCACTGGATACGACCAGACT; mmu-miR-205-5p-F: TCCTTCATTCCACCGG; mmu-miR-145a-5p-RT: GTCGTATCCAGTGCAGGGTCCGAGGTATTCGCACTGGATACGACAGGGAT; mmu-miR-145a-5p-F: GTCCAGTTTTCCCAGGA; mmu-miR-212-5p-RT: GTCGTATCCAGTGCAGGGTCCGAGGTATTCGCACTGGATACGACAGTAAG; mmu-miR-212-5p-F: ACCTTGGCTCTAGACTG; mmu-miR-139-5p-RT: GTCGTATCCAGTGCAGGGTCCGAGGTATTCGCACTGGATACGACCTGGAG; mmu-miR-139-5p-F: TCTACAGTGCACGTGT; mmu-miR-196-5p-RT: GTCGTATCCAGTGCAGGGTCCGAGGTATTCGCACTGGATACGACCCCAAC; mmu-miR-196-5p-F: TAGGTAGTTTCATGTT; Universe-R: GTGCAGGGTCCGAGGT; U6-F: CTCGCTTCGGCAGCACA; U6-R: AACGCTTCACGAATTTGCGT; Homer2-F: CACGTACCTTCCCCTTGGTG; Homer2-R: AGGGTTCGGAGAAACAGAGG; Smad3-F: GTGCGGAAACCCAAACTTTCT; Smad3-R: TAACTCTGGAGAACTTGCCCG; Homer1-F: AAGTCGCAGGAGAAGATGGAGC; Homer1-R: GGTGTTCTCTCATCGTCTGTCC; Ccnd2-F: GCAGAAGGACATCCAACCGTAC; Ccnd2-R: ACTCCAGCCAAGAAACGGTCCA; Col4a1-F: ATGGCTTGCCTGGAGAGATAGG; Col4a1-R:TGGTTGCCCTTTGAGTCCTGGA.

Droplet digital PCR was performed according to a previous study [28]. The probe sequences were as follows: mmu-miR-205-5p: 5′-VIC-AGTCTGGTCGTATCCAGTGCG-BHQ1-3′; mmu-miR-145a-5p: 5′-VIC-ATCCCTGTCGTATCCAGTGCG-BHQ1-3′; mmu-miR-212-5p: 5′-VIC-CTTACTGTCGTATCCAGTGCG-BHQ1-3′; mmu-miR-139-5p: 5′-VIC-CTCCAGGTCGTATCCAGTGCG-BHQ1-3′; mmu-miR-196-5p: 5′-VIC-TTGGGGTCGTATCCAGTGCG-BHQ1-3′.

### Western Blotting

Total protein samples were prepared from cultured P19-derived neurons or brain tissue using a radio immunoprecipitation assay buffer (#R0010; Solarbio Life Science, Beijing, China) according to the manufacturer’s instructions. Bicinchoninic acid assay was used to measure the protein concentration. Approximately 30 μg of total protein was boiled in loading buffer for 5 min at 100 °C, separated using 10–12% sodium dodecyl sulfate (SDS)–PAGE, and transferred onto polyvinylidene difluoride (PVDF) membranes (Merck Millipore, Burlington, MA, USA). Subsequently, the PVDF membranes were incubated in 5% bovine serum albumin for 2–3 h at room temperature, incubated overnight in diluted primary antibodies at 4 °C, washed with Tris-buffered saline with 0.1% Tween® 20 detergent, and incubated again in horseradish peroxidase-conjugated secondary antibodies for 1–2 h at room temperature. Next, protein bands were developed using enhanced electrochemiluminescence substrates (#32,106; Thermo Fishier Scientific), and protein band intensities were scanned to compare protein abundance.

The primary antibodies used were anti-Ndufa4 (#ab129752; Abcam), anti-B cell lymphoma (Bcl2)-associated X protein (Bax, #50,599–2; ProteinTech, Rosemont, IL, USA), anti-Bcl-2 (#bs-0032; Bioss Antibodies, Woburn, MA, USA), anti-B cell lymphoma-extra-large (Bcl-XL, #ab32370; Abcam), anti-caspase3 (#4051; Abcam), anti-caspase-9 (#7885; Santa Cruz Biotechnology, Dallas, TX, USA), anti-Homer1 (#12,433–1-AP; ProteinTech), anti-Ccnd2 (#ab207604; Abcam), and anti-Gapdh (#60,004–1-lg; ProteinTech).

### Dual Luciferase Reporter Assay

The direct binding of mmu-miR-145a-5p with the *Ndufa4*, *Homer1*, and *Ccnd2* 3′ UTRs in P19-derived neurons were separately validated using dual luciferase reporter assay with the Dual Luciferase Reporter Assay kit (#E1910; Promega Corporation). Briefly, the *Ndufa4*, *Homer1*, and *Ccnd2* 3′ UTRs were ligated with the pmirGLO vector. Next, the recombinant vectors were transfected into cultured P19-derived neurons, together with mmu-miR-145a-5p mimics or their negative control sequences, as designated using Lipofectamine 2000 Reagent (Thermo Fisher Scientific) according to the manufacturer’s instructions. Finally, the P19-derived neurons were lysed using passive lysis buffer and their luciferase activity was measured using a GloMax-20/20 luminometer (Promega Corporation).

### Statistical Analysis

Quantitative data from at least biological replicates were analyzed using SPSS Statistics version 20.0 (IBM Corporation, Armonk, NY, USA) and presented as means ± standard deviation (SD). Student’s *t*-tests were performed to define significant differences between two or more groups, and *P* < 0.05 was considered statistically significant.

## Results

### *Ndufa4* Depletion Causes Abnormal Histologic and Cellular Alterations in the Mouse Brain Tissue

**Fig. 1:**
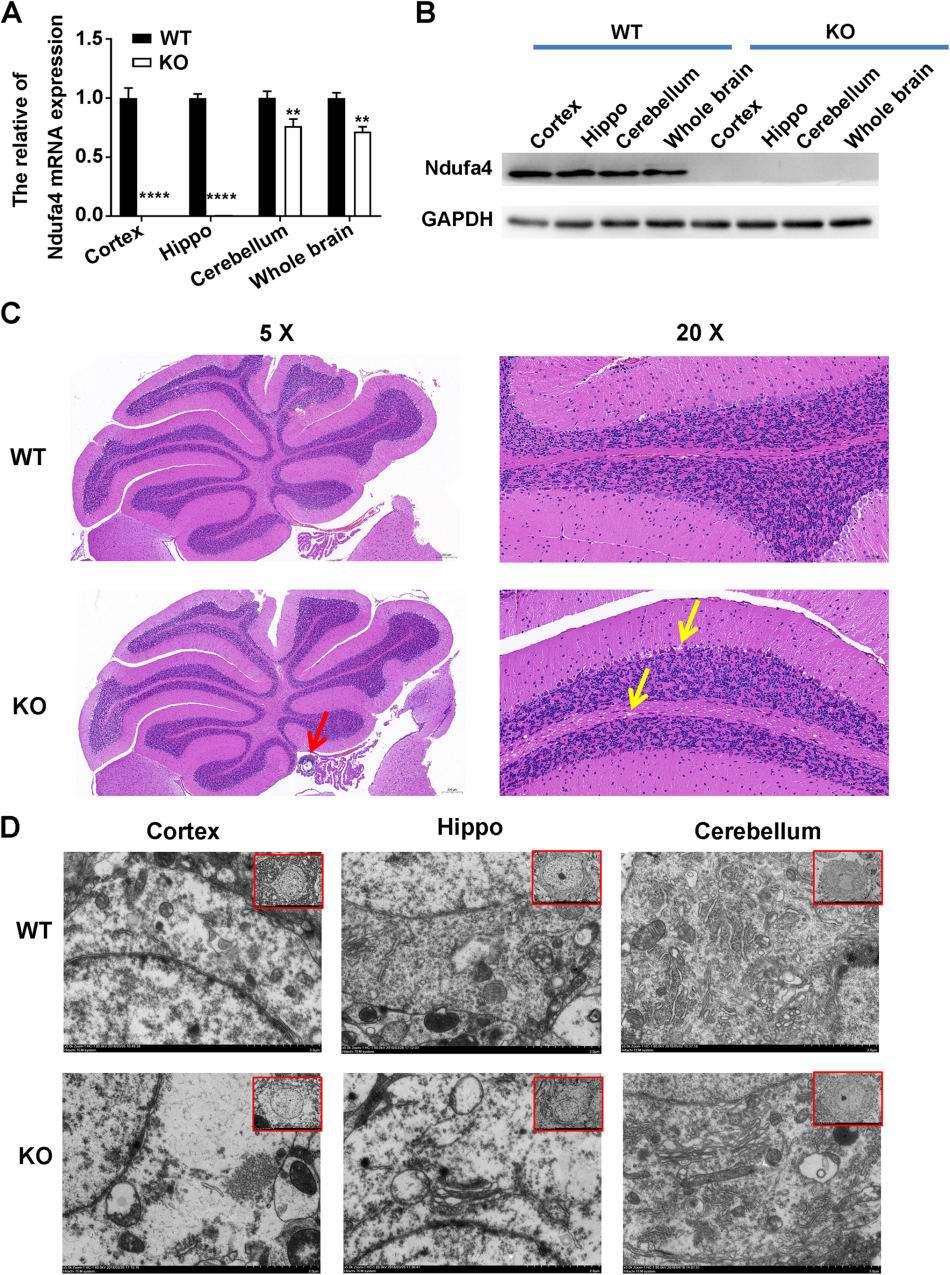
Abnormal brain tissue histological structures and cellular alterations in *Ndufa4*-KO mice. (**A**) Decreased Ndufa4 mRNA expression levels in the cortex, hippocampus, cerebellum, and whole brain of *Ndufa4*-KO mice. Real-time quantitative polymerase chain reaction was performed to analyze Ndufa4 mRNA levels. (**B**) Significant downregulation of Ndufa4 in the cortex, hippocampus, cerebellum, and whole brain of *Ndufa4*-KO mice. Western blotting was used to detect Ndufa4 levels in brain tissue. (**C**) Alterations of histological structures of the cerebellum induced by *Ndufa4* KO. The cerebellum of *Ndufa4*-KO mice was subjected to H&E staining and observed under a microscope. Enhanced basophilia in Purkinje cells and presentation of white calcification nodules are highlighted by red and yellow assays, respectively. (**D**) Cellular alterations in neurons in the cortex, hippocampus, and cerebellum of *Ndufa4*-KO mice. Subcellular organelle destruction and mitochondrial damage of neurons in *Ndufa4*-KO mice were observed by TEM. **P < 0.01, and ****P < 0.0001. WT, wild-type; KO, knockout; Ndufa4, NADH dehydrogenase (ubiquinone) 1 alpha subcomplex 4; GAPDH, glyceraldehyde-3-phosphate dehydrogenase; mRNA, messenger RNA; H&E, hematoxylin and eosin; TEM, transmission electron microscopy

To study the role of *Ndufa4* in neurons in vivo, *Ndufa4*-KO mice were established using the Cre/LoxP system. Real time-qPCR showed that the Ndufa4 mRNA levels in the cortex, hippocampus, cerebellum, and whole brain were significantly lower in the *Ndufa4*-KO mice compared with wild-type (WT) mice (Fig. 1A and Supplemental Figure S2 A). Western blotting results showed that *Ndufa4* KO significantly decreased NDUFA4 protein levels in the cortex, hippocampus, cerebellum, and whole brain (Fig. 1B). H&E staining showed normal histological structures of the cerebellum in WT mice, including a regular cell lining of the cerebellum molecular layer, granular cell layer, and Purkinje cells (Fig. 1C). However, compared with WT mice, *Ndufa4*-KO mice showed enhanced basophilia in the Purkinje cells and substantial white calcification nodules (Fig. 1C). In addition, TEM analysis showed massive neuronal edema and swelling in the cortex, in addition to considerable plasma membrane destruction, decreased organelle density, severe mitochondria damage, and rough endoplasmic reticulum extension (Fig. 1D). Similar neuronal edema, mitochondria damage, and subcellular organelle destruction was observed in the hippocampus and cerebellum of *Ndufa4*-KO mice compared with WT mice (Fig. 1D). These results indicated that *Ndufa4* expression is critical for maintaining normal histological structures and mitochondrial functions in the mouse cortex, hippocampus, and cerebellum.

### *Ndufa4* Depletion Impairs the Spatial Learning Capacity and Exploratory Activity in Mice

**Fig. 2:**
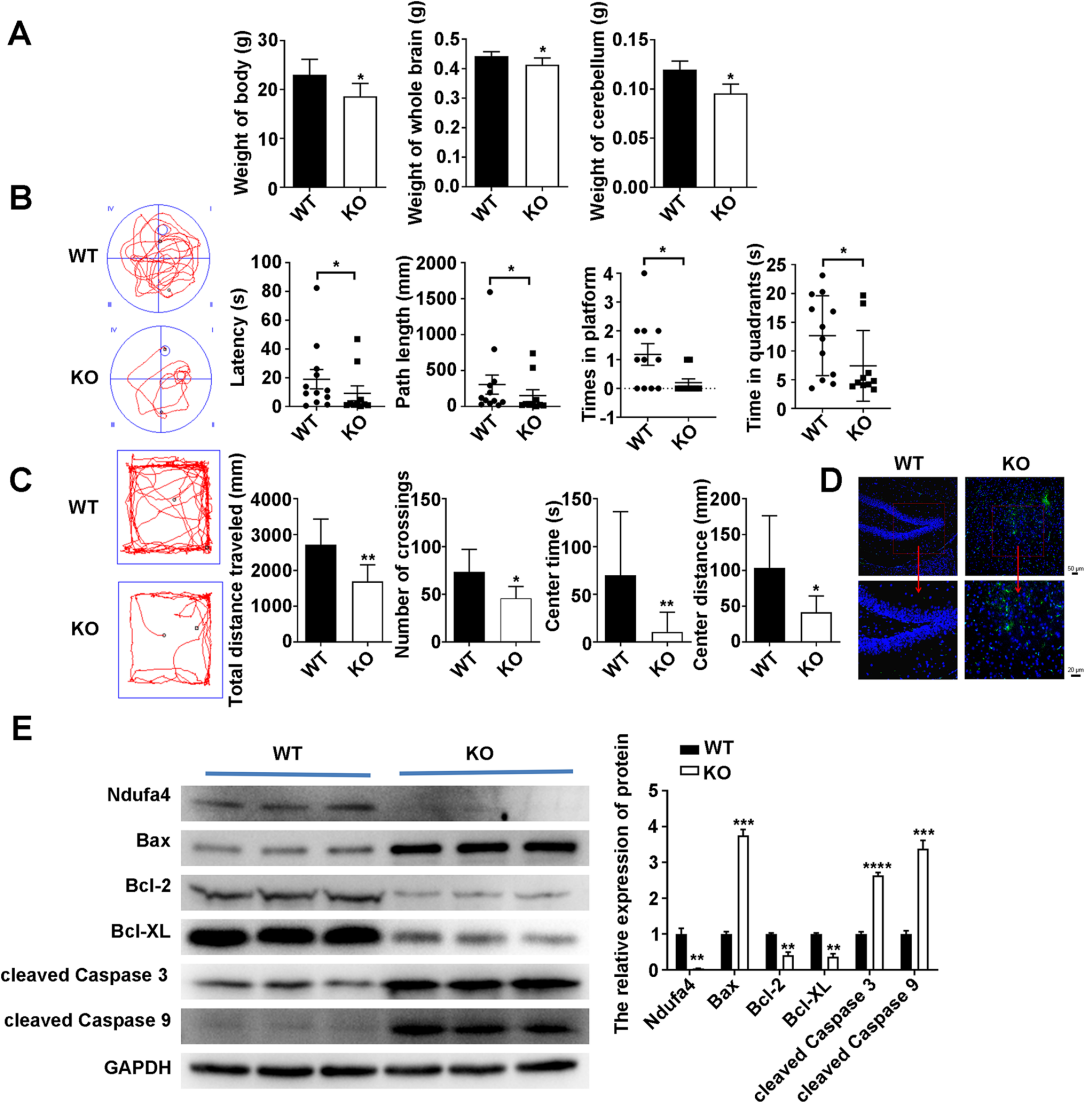
Impaired mouse behaviors and promotion of cerebellum neuron apoptosis in *Ndufa4*-KO mice. (**A**) Decrease in body, whole brain, and cerebellum weights in *Ndufa4*-KO mice. (**B**) Effects of *Ndufa4* KO on the spatial learning capacity. The spatial learning capacity of WT mice and *Ndufa4*-KO mice was evaluated using the Morris water maze test. (**C**) Inhibition of exploratory activity caused by *Ndufa4* KO. The exploratory activity of mice was assessed by the open-field test. (**D**) The apoptosis was evaluated caused by *Ndufa4* KO. The apoptosis of brain tissues was evaluated by terminal deoxynucleotidyl transferase-mediated dUTP-biotin nick end labeling assay. (**E**) Changes in Ndufa4 and apoptosis-related protein levels in the tissues of *Ndufa4*-KO mice. Western blotting was used to detect protein levels in neurons, with GAPDH as the internal standard. *P < 0.05, **P < 0.01, ***P < 0.001, and ****P < 0.0001. WT, wild-type; KO, knockout; Ndufa4, NADH dehydrogenase (ubiquinone) 1 alpha subcomplex 4; Bcl-2, B-cell lymphoma 2; Bax, Bcl2-associated X; Bcl-XL, B-cell lymphoma-extra-large; GAPDH, glyceraldehyde-3-phosphate dehydrogenase

The body, whole brain, and cerebellum weights of *Ndufa4*-KO mice were slightly lower than those of WT mice (Fig. 2A). To gain a better understanding of the physiological functions of *Ndufa4*, the behavioral alterations of *Ndufa4*-KO mice were compared with those of WT mice. The Morris water maze test showed that the latency time, path length, times on the platform, and time in quadrants were lower for *Ndufa4*-KO mice than for WT mice, indicating that *Ndufa4* depletion impairs the spatial learning capacity (Fig. 2B), although it could not completely rule out the influence of loss of respiratory chain components caused by the whole body knockout. Similarly, the open-field test showed decreased total distance traveled, number of crossings, center distance, and center time in *Ndufa4*-KO mice, indicating poor exploratory activity (Fig. 2C). Furthermore, TUNEL staining showed increased apoptosis in the brain tissues of *Ndufa4*-KO mice compared with WT mice (Fig. 2D). In addition, western blotting showed that NDUFA4, Bcl-2, and Bcl-XL levels in the cerebellum considerably decreased in *Ndufa4*-KO mice compared with WT mice (Fig. 2E). In contrast, *Ndufa4* KO significantly increased Bax, cleaved caspase-3, and cleaved caspase-9 levels were observed in in the cerebellum of *Ndufa4*-KO mice (Fig. 2E). These results showed that *Ndufa4* KO causes apoptosis of the neurons in the cerebellum and substantially impairs mouse behaviors.

### *Ndufa4* Overexpression Promotes Neuronal Proliferation and Inhibits Neuron Apoptosis, Whereas *Ndufa4* KO Has the Opposite Effect in P19-Derived Neurons

**Fig. 3:**
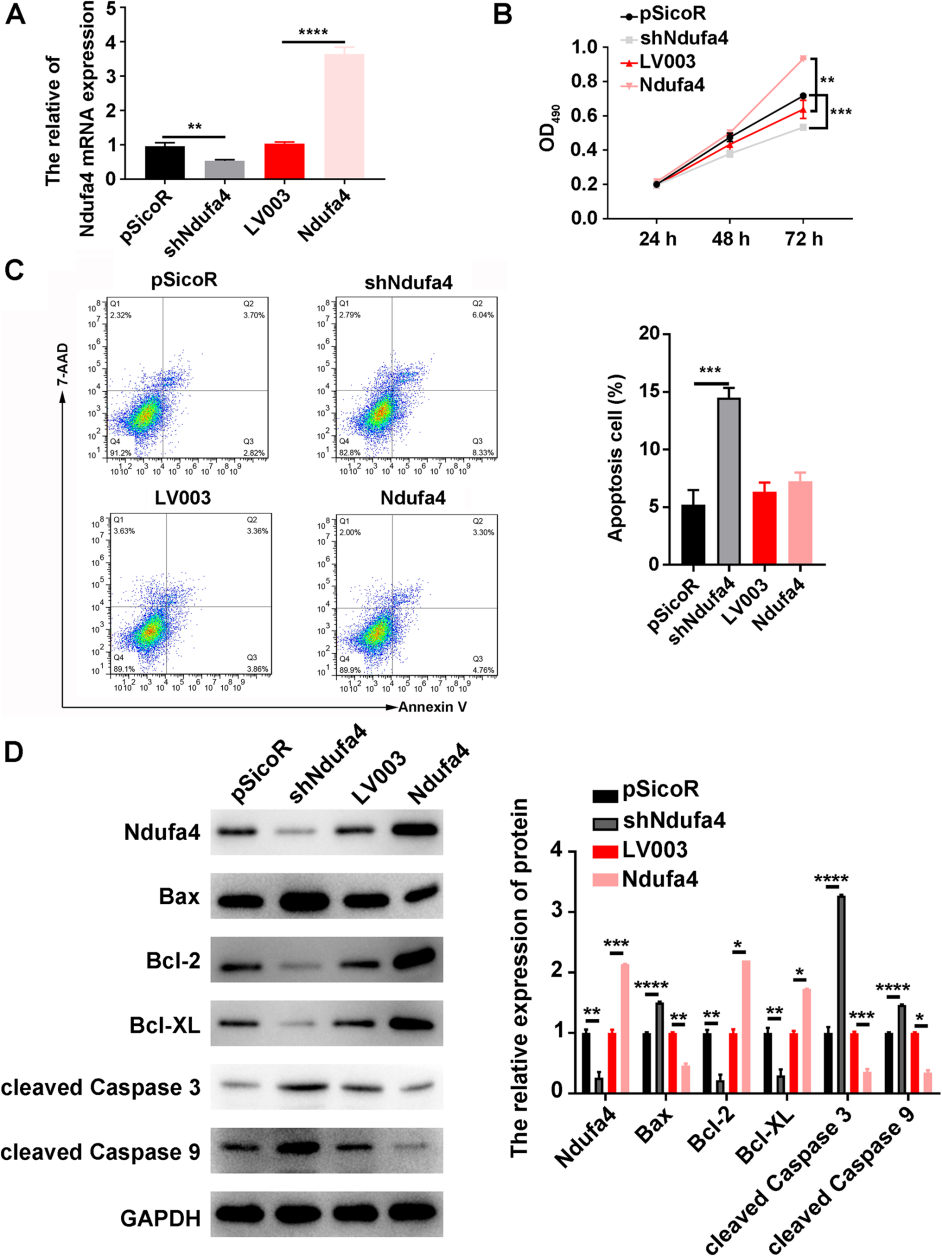
Modulation of neuronal proliferation and apoptosis by *Ndufa4* KO and overexpression. (**A**) Relative Ndufa4 mRNA expression in P19-derived neurons transfected with shNdufa4 or LV003-Ndufa4 (Ndufa4) vectors. Real time-quantitative polymerase chain reaction was used to detect Ndufa4 mRNA levels. (**B**) Alterations of P19-derived neuronal proliferation after *Ndufa4* KO and overexpression. Neuronal proliferation was assessed using MTS assay. (**C**) Regulation of P19-derived neuronal apoptosis by *Ndufa4* KO and overexpression. The percentages of apoptotic neurons were measured by flow cytometry. (**D**) Differential expression of Ndufa4 and apoptosis-related proteins in P19-derived neurons transfected with shNdufa4 or LV003-Ndufa4 (Ndufa4) vectors. Western blotting was performed to analyze protein levels in neurons, with GAPDH as the internal standard. *P < 0.05, **P < 0.01, ***P < 0.001, and ****P < 0.0001.Ndufa4, NADH dehydrogenase (ubiquinone) 1 alpha subcomplex 4; Bcl-2, B-cell lymphoma 2; Bax, Bcl2-associated X; Bcl-XL, B-cell lymphoma-extra-large; GAPDH, glyceraldehyde-3-phosphate dehydrogenase; KO, knockout; mRNA, messenger RNA; MTS, 3-(4,5-dimethylthiazol-2-yl)-5-(3-carboxymethoxyphenyl)-2-(4-sulfophenyl)-2H-tetrazolium

To evaluate the effects of *Ndufa4* expression on neuron functions, P19 cells were differentiated into neurons by treating them with RA as introduced in the “Materials and Methods” section. Subsequently, the *Ndufa4* expression in induced neurons was altered by transfection with *shNdufa4* or LV003-*Ndufa4* (*Ndufa4*). Real-time qPCR showed that *shNdufa4* was considerably inhibited, but LV0003-*Ndufa4* vectors increased *Ndufa4* mRNA expression in P19-derived neurons (Fig. 3A and Supplemental Figure S2 B). The MTS assay showed that *shNdufa4* was considerably decreased, but *Ndufa4* overexpression considerably promoted the proliferation of P19-derived neurons compared with the control group (Fig. 3B). Conversely, *shNdufa4* was considerably promoted, but *Ndufa4* overexpression considerably inhibited neuron apoptosis in P19-derived neurons (Fig. 3C). Consistent with this result, western blotting showed that *shNdufa4* was subbstantially decreased, but *Ndufa4* overexpression substantially increased NDUFA4, Bcl-2, and Bcl-XL levels in P19-derived neurons (Fig. 3D). In addition, *shNdufa4* was substantially upregulated, but *Ndufa4* overexpression substantially downregulated the Bax, cleaved caspase-3, and cleaved caspase-9 levels in P19-derived neurons (Fig. 3D). These results indicated that Ndufa4 overexpression promoted the proliferation and inhibited the apoptosis of P19-derived neurons. After knockout or overexpression of Ndufa4 in mouse neural stem cell line NE-4C, cell proliferation is also inhibited or promoted. (Supplemental Figure S3).

### *Ndufa4* Modulates microRNA Expression and Inhibits miR-212-5p and miR-145a-5p in the Cerebellum and Neurons

**Fig. 4:**
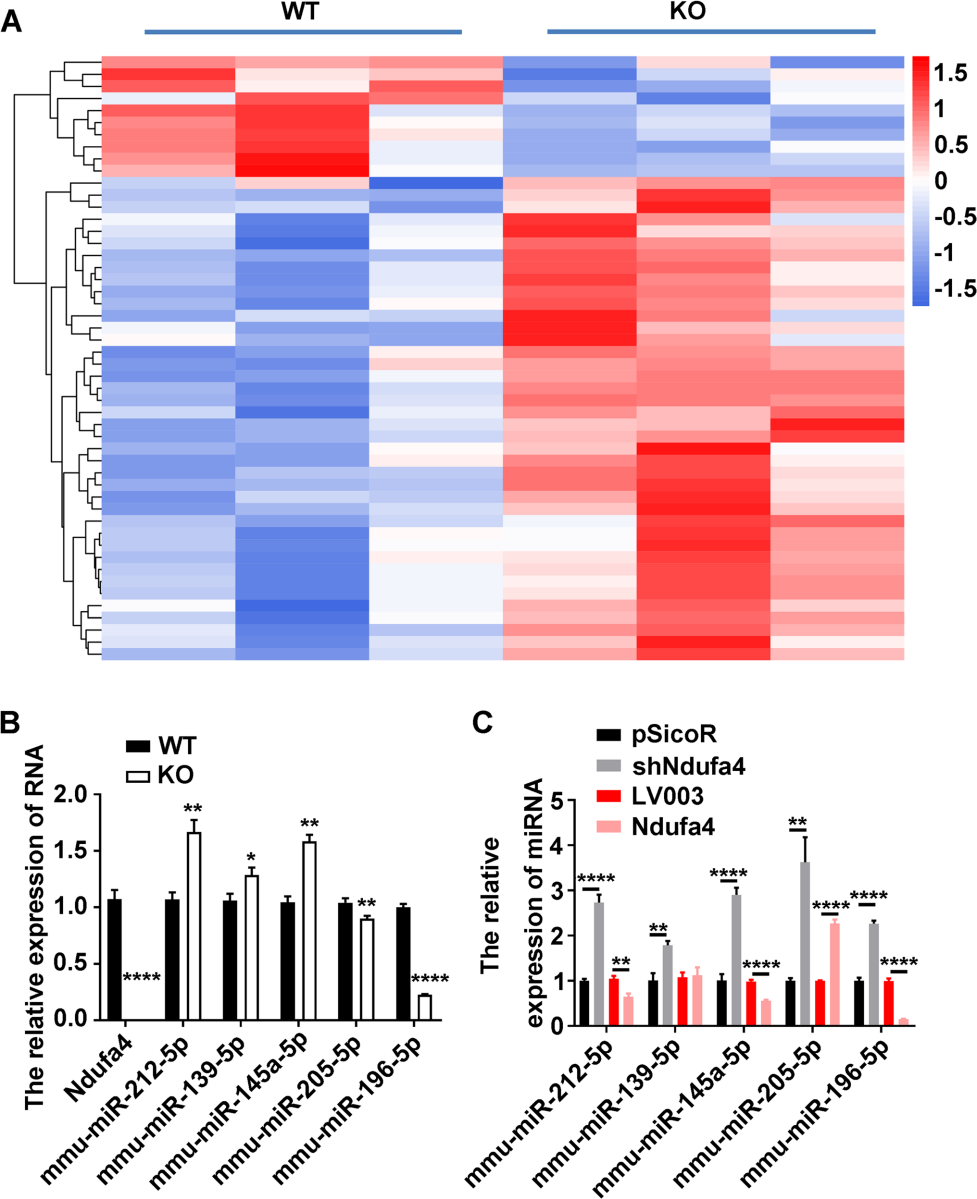
Differential microRNA expression induced by *Ndufa4* KO in mouse cerebellum and neurons. (**A**) Hierarchy clustering of differentially expressed microRNAs in the cerebellum of *Ndufa4*-KO mice. Differentially expressed microRNAs were characterized by next-generation deep sequencing (FC >  = 1.5; *P* < 0.05). The increased and downregulated microRNA expression is indicated by red and blue bars, respectively. (**B**) Relative microRNA expression in the cerebellum of *Ndufa4*-KO mice. Relative microRNA expression in the cerebellum was detected by real time-quantitative polymerase chain reaction. (**C**) Differential expression of candidate microRNAs in P19-derived neurons transfected with shNdufa4 or LV003-Ndufa4 (Ndufa4) vectors. Real time-quantitative polymerase chain reaction was conducted to measure microRNA expression. *P < 0.05, **P < 0.01, and ****P < 0.0001. WT, wild-type; KO, knockout; Ndufa4, NADH dehydrogenase (ubiquinone) 1 alpha subcomplex 4; FC, fold-change

To determine the molecular mechanism underlying *Ndufa4* functions, a large-scale identification of differentially expressed microRNAs in the cerebellum was conducted between WT mice and *Ndufa4*-KO mice using next-generation deep sequencing, and the potential mechanism of *Ndufa4* in the cortex and hippocampus will be shown in a future study. In all, 50 microRNAs were differentially expressed between the WT and *Ndufa4*-KO mice cerebellum. Of these, 40 were upregulated and 10 downregulated in *Ndufa4*-KO mice (FC >  = 1.5; Fig. 4A). Real-time qPCR and droplet digital PCR showed that mmu-miR-212-5p, mmu-miR-139a-5p, and mmu-miR-145a-5p expression considerably increased; however, mmu-miR-205a-5p and mmu-miR-196-5p expression considerably decreased in the cerebellum of *Ndufa4*-KO mice (Fig. 4B and Supplemental Figure S4 A). In addition, *shNdufa4* considerably increased but *Ndufa4* overexpression inhibited mmu-miR-212-5p, mmu-miR-145a-5p, and mmu-miR-196-5p expression in P19-derived neurons (Fig. 4C and Supplemental Figure S4 B). These results indicated that *Ndufa4* regulated neuronal functions and mouse brain development and behaviors by inhibiting downstream microRNAs such as mmu-miR-212-5p and mmu-miR-145a-5p.

### *miR-145a-5p* Inhibits the Proliferation of Neurons and Promotes Their Apoptosis

**Fig. 5:**
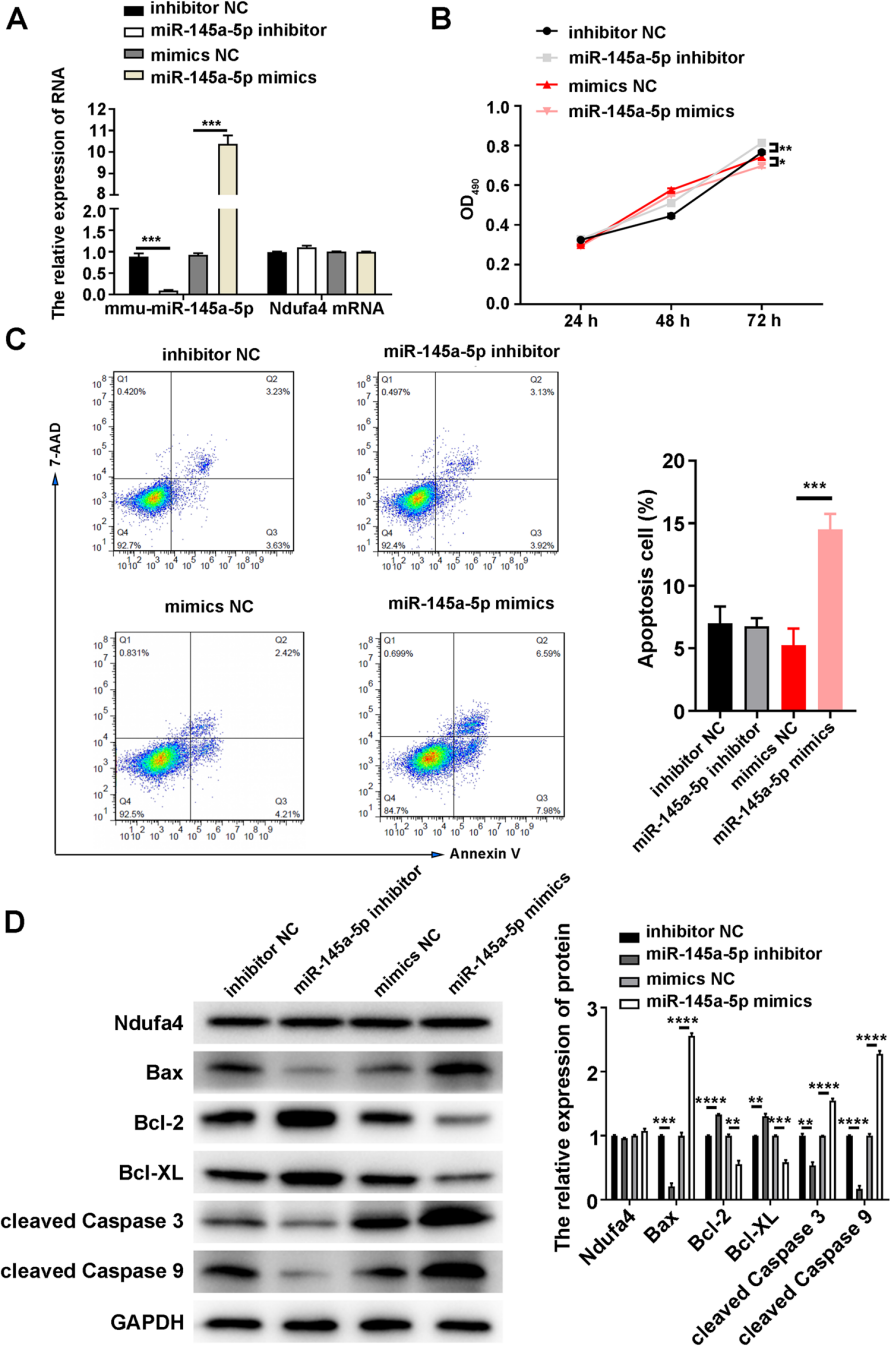
Regulation of neuronal proliferation and apoptosis by miR-145a-5p. (**A**) Relative miR-145a-5p and Ndufa4 mRNA expression in P19-derived neurons transfected with miR-145a-5p inhibitors or mimics. The mRNA and microRNA levels in neurons were detected by real time-quantitative polymerase chain reaction. (**B**) Alteration in neuronal proliferation rates induced by miR-145a-5p inhibitors or mimics. MTS assay was used to analyze the proliferation of P19-derived neurons. (**C**) Elevated percentages of apoptotic neurons owing to miR-145a-5p mimics. The apoptosis of P19-derived neurons was assessed by flow cytometry. (**D**) Effects of miR-145a-5p inhibitors or mimics on the levels of Ndufa4 and apoptosis-related proteins in P19-derived neurons. Protein levels in neurons were analyzed by western blotting, with GAPDH as the internal standard. *P < 0.05, **P < 0.01, ***P < 0.001, and ****P < 0.0001. RA, all‐trans‐retinoid acid; NC, negative control; Ndufa4, NADH dehydrogenase (ubiquinone) 1 alpha subcomplex 4; OD490, optical density at 490 nm; Bcl-2, B-cell lymphoma 2; Bax, Bcl2-associated X; Bcl-XL, B-cell lymphoma-extra-large; GAPDH, glyceraldehyde-3-phosphate dehydrogenase; mRNA, messenger RNA; MTS, 3-(4,5-dimethylthiazol-2-yl)-5-(3-carboxymethoxyphenyl)-2-(4-sulfophenyl)-2H-tetrazolium

For cellular function analysis of miR-145a-5p, the miR-145a-5p expression in P19-derived neurons was altered by transfection with miR-145a-5p inhibitors or mimics. Real time-qPCR and droplet digital PCR confirmed that miR-145a-5p inhibitors considerably downregulated but miR-145a-5p mimics upregulated the miR-145a-5p expression in neurons (Fig. 5A and Supplemental Figure S4 C). Alterations in miR-145a-5p expression did not induce any change in the *Ndufa4* mRNA content in P19-derived neurons (Fig. 5A and Supplemental Figure S2 C). The MTS assay showed that miR-145a-5p inhibitors substantially promoted but miR-145a-5p mimics substantially inhibited the proliferation of P19-derived neurons compared with negative controls (Fig. 5B). Flow cytometry showed that miR-145a-5p mimics substantially increased but miR-145a-5p inhibitors did not affect the percentage of apoptotic P19-derived neurons compared with the control group (Fig. 5C). Consistent with this result, miR-145a-5p inhibitors substantially increased but miR-145a-5p mimics substantially decreased Bcl-2 and Bcl-XL levels in P19-derived neurons (Fig. 5D). However, completely opposite alterations in Bax, cleaved caspase-3, and cleaved caspase-9 were observed in P19-derived neurons transfected with miR-145a-5p inhibitors or mimics (Fig. 5D). NDUFA4 levels in P19-derived neurons were not affected by miR-145a-5p inhibitors or mimics (Fig. 5D). These results indicated that miR-145a-5p had considerable regulatory effects on neuronal proliferation and apoptosis.

### *Ndufa4* Promotes and miR-145a-5p Inhibits Homer1 and Ccnd2 Expression in Neurons

**Fig. 6:**
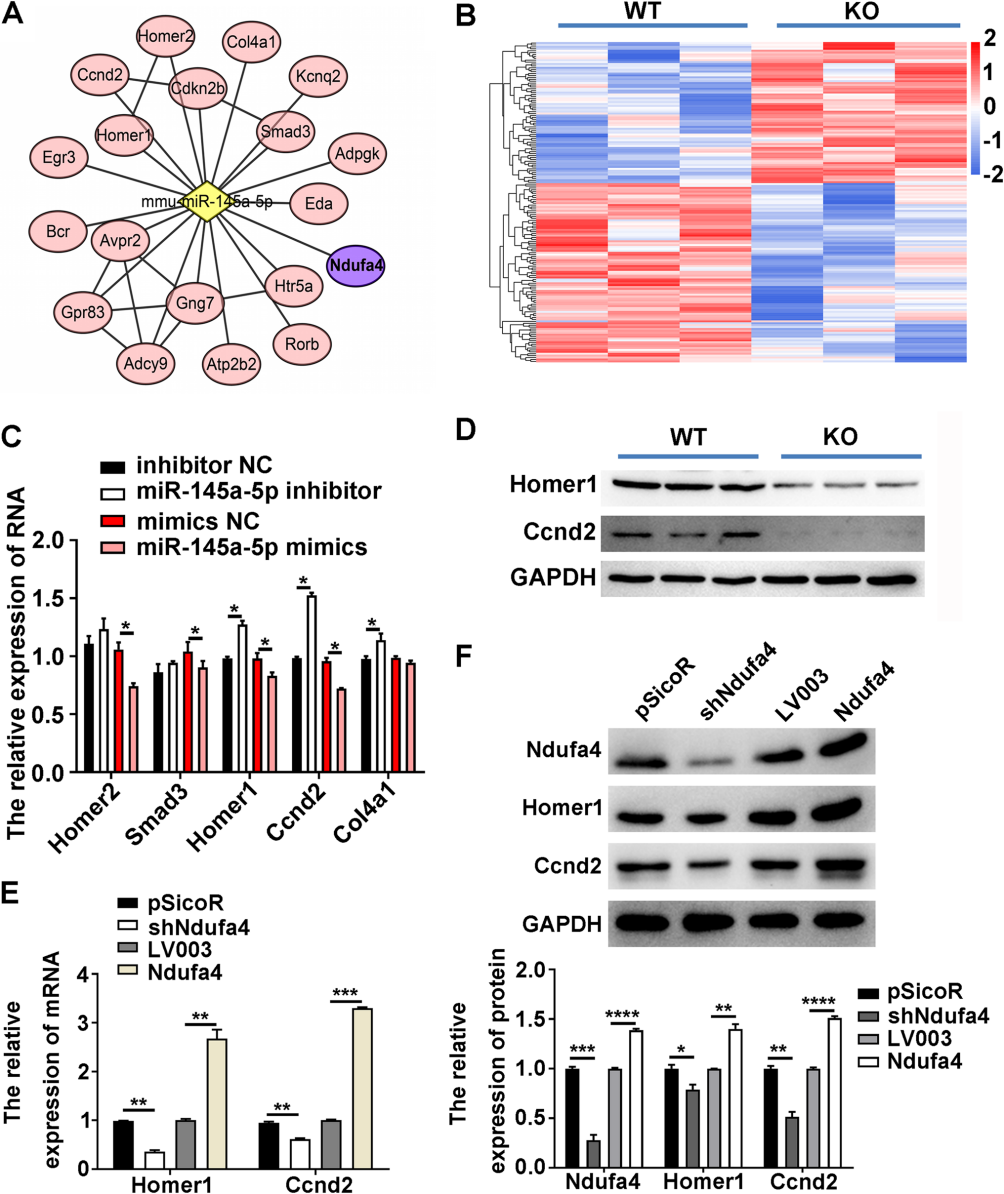
Modulation of Homer1 and Ccnd2 expression in neurons by Ndufa4 and miR-145a-5p. (**A**) Interaction between miR-145a-5p and its predicted target genes established by Targetscan software. Target genes are indicated in red or purple ovals. (**B**) Hierarchy clustering of differentially expressed mRNAs in the cerebellum of *Ndufa4*-KO mice. Differentially expressed mRNAs in tissues were characterized by next-generation deep sequencing (FC > 1.5; *P* < 0.05). Upregulated and downregulated mRNA expression is indicated by red and blue bars, respectively. (**C**) Differential Homer1, Homer2, Smad3, Col4a1, and Ccnd2 mRNA expression in P19-derived neurons transfected with miR-145a-5p inhibitors or mimics. Real time-quantitative polymerase chain reaction was used to detect mRNA levels. (**D**) Significant inhibition of Homer1 and Ccnd2 levels in the cerebellum of *Ndufa4*-KO mice. Protein levels were measured by western blotting. (**E**, **F**) Alterations in Homer1 and Ccnd2 expression in P19-derived neurons induced by shNdufa4 or Ndufa4 overexpression. Homer1 and Ccnd2 expression in neurons was detected by real time-quantitative polymerase chain reaction (**E**) and western blotting (**F**), respectively. *P < 0.05, **P < 0.01, ***P < 0.001, and ****P < 0.0001. Ndufa4, NADH dehydrogenase (ubiquinone) 1 alpha subcomplex 4; WT, wild-type; KO, knockout; RA, all‐trans‐retinoid acid; NC, negative control; Homer1/2, human homer protein homolog 1/2; Smad3, Sma- and Mad-related protein 3; Col4a1, collagen 4a1; Ccnd2, cyclin D2; GAPDH, glyceraldehyde-3-phosphate dehydrogenase; FC, fold-change; mRNA, messenger RNA

To study the downstream mechanisms underlying *Ndufa4*/miR-145a-5p-regulated neuronal functions, the potential target genes that may be regulated by *Ndufa4* and miR-145a-5p were examined. Targetscan analysis showed that multiple candidate genes, such as *Homer1*, *Ccnd2*, and *Ndufa4*, were targeted by miR-145a-5p (Fig. 6A). Subsequently, next-generation deep sequencing of differential mRNA profiles in the cerebellum of *Ndufa4*-KO mice was performed. Results showed 74 upregulated and 91 downregulated mRNAs, such as *Homer1*, *Homer2*, Sma- and Mad-related protein 3 (*Smad3*), collagen 4a1 (*Col4a1*), and *Ccnd2*, in *Ndufa4*-KO mice compared with WT mice (FC > 1.5; *P* < 0.05) (Fig. 6B). Subsequently, miR-145a-5p inhibitors considerably increased but miR-145a-5p mimics considerably inhibited Homer1 and *Ccnd2* mRNA expression in P19-derived neurons (Fig. 6C). In addition, the cerebellum of *Ndufa4*-KO mice showed significant downregulation of Homer1 and Ccnd2 levels compared with WT mice (Fig. 6D). Rela time-qPCR and western blotting showed that sh*Ndufa4* considerably inhibited but *Ndufa4* overexpression promoted Homer1 and Ccnd2 expression in P19-derived neurons (Fig. 6E, Supplemental Figure S2 D, and 6F). These results showed that miR-145a-5p inhibited and *Ndufa4* promoted Homer1 and Ccnd2 expression in P19-derived neurons.

### Ndufa4 3′ UTR Inhibits miR-145a-5p, Increases Homer1 and Ccnd2, and Increases Neuronal Proliferation

**Fig. 7:**
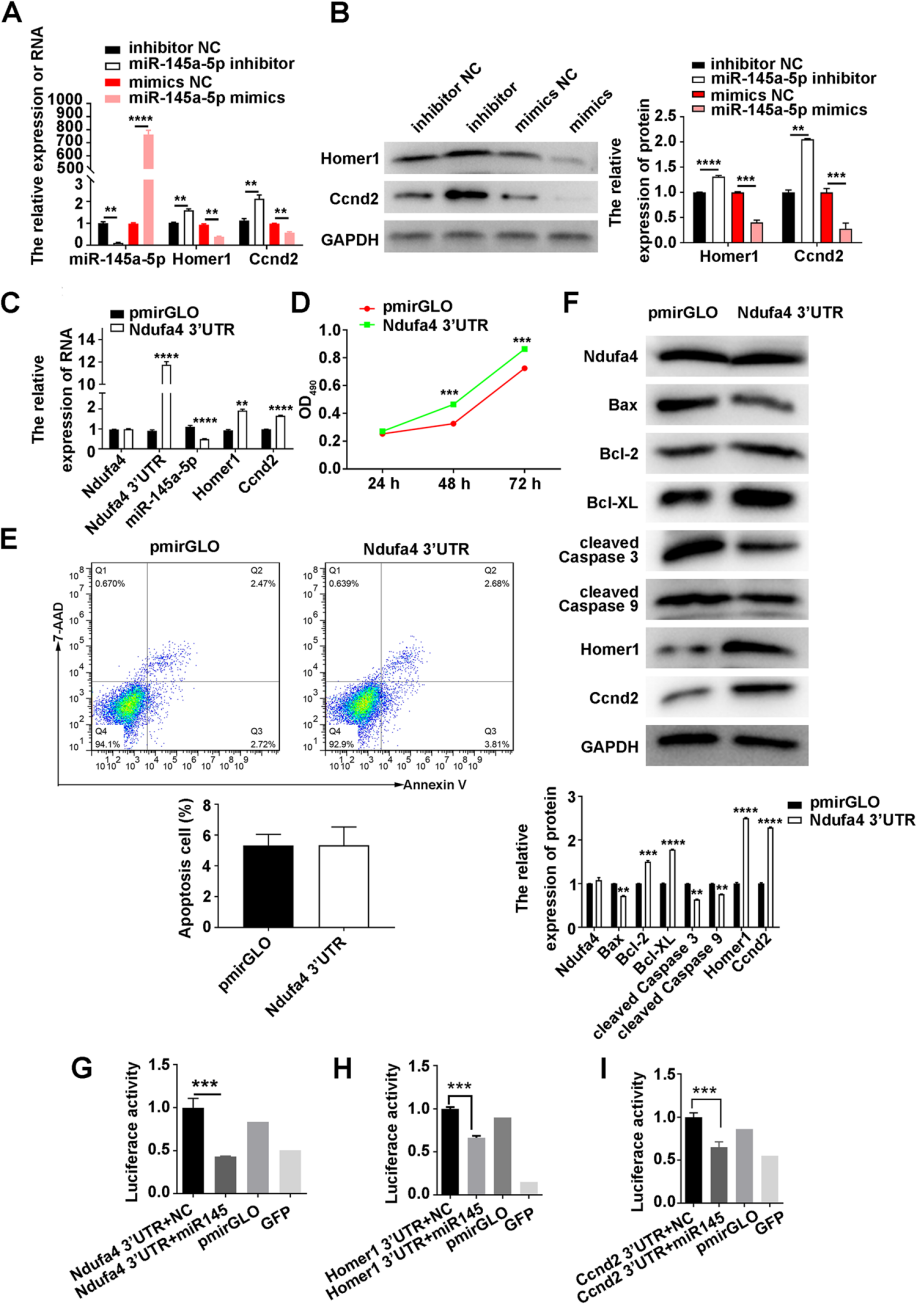
Modulation of miR-145a-5p, Homer1, and Ccnd2 expression and neuronal proliferation by *Ndufa4* 3′ UTR overexpression. (**A**, **B**) Alterations in Homer1 and Ccnd2 expression in P19-derived neurons, induced by miR-145a-5p inhibitors or mimics. Homer1 and Ccnd2 mRNA and protein levels in neurons were detected by real-time quantitative polymerase chain reaction (**A**) and western blotting (**B**), respectively. (**C**) Relative expression levels of Ndufa4, *Ndufa4* 3′ UTR, miR-145a-5p, Homer1, and Ccnd2 mRNA in neurons with *Ndufa4* 3′ UTR overexpression. The mRNA and microRNA levels were quantitated by real-time quantitative polymerase chain reaction. (**D**) Enhanced proliferation rates of P19-derived neurons caused by *Ndufa4* 3′ UTR overexpression. Neuronal proliferation was evaluated by MTS assay. (**E**) Percentages of apoptotic P19-derived neurons overexpressing the *Ndufa4* 3′ UTR. Neuron apoptosis was assessed by flow cytometry. (**F**) Alteration in Ndufa4, apoptosis-related proteins, and Homer1 and Ccnd2 levels in neurons overexpressing the *Ndufa4* 3′ UTR. The direct binding of miR-145a-5p to the 3′ UTR sequences of *Ndufa4* (**G**), *Homer1* (H), and *Ccnd2* (I) was validated by the dual luciferase reporter assay. P19-derived neurons expressing *Ndufa4*, *Homer1*, and *Ccnd2* 3′ UTRs were transfected with miR-145a-5p mimics or negative controls, followed by measurement of luciferase activity. Protein levels were measured by western blotting, with GAPDH as the internal standard. **P < 0.01, ***P < 0.001, and ****P < 0.0001. RA, all‐trans‐retinoid acid; NC, negative control; Homer1, human homer protein homolog 1; Ccnd2, cyclin D2; Gapdh, glyceraldehyde-3-phosphate dehydrogenase; Ndufa4, NADH dehydrogenase (ubiquinone) 1 alpha subcomplex 4; UTR, untranslated region; Bcl-2, B-cell lymphoma 2; Bax, Bcl2-associated X; Bcl-XL, B-cell lymphoma-extra-large; Ndufa4, NADH dehydrogenase (ubiquinone) 1 alpha subcomplex 4; GFP, green fluorescence protein; NC, negative control; MTS, 3-(4,5-dimethylthiazol-2-yl)-5-(3-carboxymethoxyphenyl)-2-(4-sulfophenyl)-2H-tetrazolium; UTR, untranslated region; Homer1, human homer protein homolog 1; Ccnd2, cyclin D2

Real time-qPCR and western blotting further confirmed the miR-145a-5p-induced negative regulation of Homer1 and Ccnd2 mRNA and protein levels in P19-derived neurons (Fig. 7A, Supplemental Figure S2 E, and 7B). To assess the mechanisms underlying miR-145a-5p inhibition by *Ndufa4*, the *Ndufa4* 3**′** UTR in P19-derived neurons were overexpressed. Results showed that *Ndufa4* 3**′** UTR overexpression could considerably decrease miR-145a-5p levels and increase *Homer1* and *Ccnd2* mRNA levels in P19-derived neurons compared with neurons transfected with empty vectors (Fig. 7C, Supplemental Figure S2 F, and Supplemental Figure S4 D). In addition, *Ndufa4* 3**′** UTR overexpression substantially increased the proliferation of P19-derived neurons (Fig. 7D). Flow cytometry showed that *Ndufa4* 3**′** UTR overexpression did not considerably alter the percentage of apoptotic P19-derived neurons (Fig. 7E). However, *Ndufa4* 3**′** UTR overexpression was found to considerably decrease Bax, cleaved caspase-3, and cleaved caspase-9 levels and increase Bcl-2, Bcl-XL, Homer1, and Ccnd2 levels in P19-derived neurons (Fig. 7F). These findings showed that *Ndufa4* 3**′** UTR sequences could inhibit miR-145a-5p, increase *Homer1* and *Ccnd2* levels, and promote neuronal proliferation.

### miR-145a-5p Directly Targets *Ndufa4, Homer1, *and *Ccnd2 3*′ UTRs in Neurons

The dual luciferase reporter assay showed that transfection with miR-145a-5p mimics could considerably decrease the luciferase activity of P19-derived neurons expressing the *Ndufa4* 3**′** UTR (Fig. 7G). In addition, miR-145a-5p mimics were found to considerably decrease the luciferase activity of P19-derived neurons expressing the *Homer1* 3**′** UTR (Fig. 7H). Similarly, transfection with miR-145a-5p mimics substantially decreased the luciferase activity of P19-derived neurons expressing the *Ccnd2* 3**′** UTR compared with transfection with the negative control (Fig. 7I). These results demonstrated the direct association of miR-145a-5p with *Ndufa4*, *Homer1*, and *Ccnd2* 3**′** UTRs in P19-derived neurons and suggested that Ndufa4 promoted the proliferation of neurons and inhibited their apoptosis by inhibiting miR-145a-5p expression, which inhibits *Homer1* and *Ccnd2* expression by directly targeting their 3**′** UTRs.

## Discussion

Neuronal function dysregulation and the resultant vermian developmental arrest and fourth ventricle foramina fenestration failure are major clinical manifestations in patients with DWM [1, 7]. However, the molecular mechanisms underlying the regulation of neuronal functional alterations, such as proliferation and apoptosis, during the pathogenesis of DWM are unclear. NDUFA4 is an essential subunit of the mitochondrial respiratory chain associated with neuronal functions and various neurological disorders [16, 19]. Our previous studies showed that *NDUFA4* was closely implicated in the development of DWM [19–21]. This study used the P19-derived cellular neuron model to show that *Ndufa4* effectively promotes the proliferation of neurons and inhibits their apoptosis. *Ndufa4*-KO substantially impaired the histological structures and cellular functions of the brain, inducing considerable impairment in the spatial learning capacity and exploratory activity of mice. In addition, *Ndufa4* inhibited miR-145a-5p expression, which in turn inhibited the proliferation of neurons and promoted their apoptosis. The roles of *Ndufa4* and miR-145a-5p in regulating neuronal functions are mediated by their targeting of *Homer1* and *Ccnd2* through the direct binding of miR-145a-5p to *Homer1* and *Ccnd2* 3′ UTRs. Overall, our results provided a novel insight into the neuronal function regulation mediated by the *Ndufa4*/miR-145a5p/target gene axis.

The pathogenesis of DWM is closely associated with mutation and expressional alterations in various functional genes, such as forkhead transcription factor (*FOXC1*), fibroblast growth factor 17 (*FGF17*), and *Ndufa4* [19, 20, 29]. However, their pathogenic roles in DWM development are still unclear owing to a lack of extensive functional investigations. The present study elucidated the neuron-regulating roles of NDUFA4 by silencing or overexpressing *Ndufa4* in neurons induced from P19 cells using RA treatment, which showed substantial proliferation-promoting and apoptosis-inhibiting functions of NDUFA4 in neurons. Using *Ndufa4*-KO mice, the contribution of NDUFA4 to maintaining structural homeostasis in the brain, spatial learning capacity, and exploratory activity was elucidated. Direct evidence of alterations in NDUFA4 expression alterations that cause considerable behavioral abnormalities in the mouse model was found. These cellular and animal results convincingly established *Ndufa4* as a critical regulator of neuronal development, and behavior.

*Ndufa4* mutation and expression was also implicated in other neurological disorders, such as AD, and neurological symptoms in Leigh syndrome [16, 18]. The mediating roles of NDUFA4-regulated neuronal proliferation and apoptosis in the pathogenesis of these neurological disorders should be investigated further.

MicroRNAs, an extensive type of noncoding RNA molecules with an average size of 18–25 nt, play an essential regulatory role in various biological and pathogenic processes by modulating the target gene expression [30–32]. In addition, microRNAs significantly contribute to neuron biology and various neurological diseases [33]. In this study, we found that the expression of a large set of microRNAs is regulated by *Ndufa4* KO in the mouse brain tissue. Among these, *Ndufa4* KO considerably decreases, *Ndufa4* shRNA inhibits, and *Ndufa4* overexpression in P19-derived neurons promotes miR-145a-5p expression in the cerebellum. Of note, miR-145a-5p inhibits the proliferation of neurons and promotes their apoptosis. These results indicate the critical role of *Ndufa4*-regulated miR-145a-5p expression in DWM development and other neurological diseases. Moreover, the 3′ UTR RNAs of functional genes are a key group of endogenous natural microRNA sponges that inhibit microRNA expression as competing endogenous RNAs [34, 35]. *Ndufa4* 3′ UTR overexpression can substantially decrease miR-145a-5p expression and neuronal proliferation and apoptosis. In addition, miR-145a-5p is associated with the *Ndufa4* 3′ UTR. These findings reveal a new mechanism underlying *Ndufa4*-induced miR-145a-5p inhibition, i.e., the sponging of miR-145a-5p by the *Ndufa4* 3′ UTR.

The important biological functions of microRNAs are mediated by their inhibition of functional gene expression, mainly via binding with 3′ UTRs of target genes. For instance, the pathogenic role of miR-145a-5p in nasopharyngeal carcinoma is mediated by its inhibition of NUAK Family SNF1-like Kinase 1 (NUAK1) expression [36]. In this study, next-generation deep sequencing of differentially expressed microRNAs in the cerebellum of *Ndufa4*-KO mice was performed to characterize the miR-145a-5p target genes in neurons, combined with bioinformatic prediction. Homer1, a postsynaptic density protein, is a key regulator of neuronal synaptic activity and neurological disease pathogenesis [37, 38]. In contrast, Ccnd2 is a member of the highly conserved cyclin family that regulates cell cycle progression, cell proliferation, and apoptosis in distinct contexts whose expression is considerably regulated by microRNAs [39, 40]. In this study, miR-145a-5p could effectively inhibit *Homer1* and *Ccnd2* expression in neurons, which was significantly promoted by Ndufa4 in neurons. In addition, *Ndufa4* 3′ UTR overexpression was found to considerably promote *Homer1* and *Ccnd2* expression in neurons. In addition, miR-145a-5p was found to directly bind to *Homer1* and *Ccnd2* 3′ UTRs. These results indicated that Homer1 and Ccnd2 expression was considerably regulated by *Ndufa4*/miR-145a-5p associated with neuronal functioning.

However, our research has limitations. First, the whole body knockout of Ndufa4 cannot completely avoid its influence on the behavioral phenotype of mice as a component of the respiratory chain. Regional specific deletion may be more appropriate, but at present, we are unable to obtain these mice. Second, the detection of O_2_ consumption rate and ATP generation in neurons may provide more powerful evidence for miR-145 mediated effects, which will be further explored in our future research.

In summary, the mitochondrial respiratory chain component protein NDUFA4 was found to promote the proliferation of neurons and inhibit their apoptosis by inhibiting miR-145a-5p to enhance *Homer1* and *Ccnd2* expression. The inhibitory effects of *Ndufa4* on miR-145a-5p functioning could be mediated by the direct association of the *Ndufa4* 3′ UTR with miR-145a-5p. These findings reveal novel clues regarding the neuron growth and apoptosis in embryonic development and other neurological disorders.

## Supplementary Information

Below is the link to the electronic supplementary material.

Supplemental Figure S1 The flow chart of generation of *Ndufa4*-KO mice. (PNG 1169 kb)

High resolution image (TIF 8219 kb)

Supplemental Figure S2 Real time-quantitative polymerase chain reaction results of Figure 1A, 3A, 5A, 6E, 7A, and 7C using RPL7 as reference. (A) Real time-quantitative polymerase chain reaction results of Figure 1A using RPL7 as reference. (B) Real time-quantitative polymerase chain reaction results of Figure 3A using RPL7 as reference. (C) Real time-quantitative polymerase chain reaction results of Figure 5A using RPL7 as reference. (D) Real time-quantitative polymerase chain reaction results of Figure 6E using RPL7 as reference. (E) Real time-quantitative polymerase chain reaction results of Figure 7A using RPL7 as reference. (F) Real time-quantitative polymerase chain reaction results of Figure 7C using RPL7 as reference. *P < 0.05, **P < 0.01, ***P < 0.001 and ****P < 0.0001. RPL7, ribosomal Protein L7. (PNG 476 kb)

High resolution image (TIF 3083 kb)

Supplemental Figure S3 Alterations of NE-4C neural stem cells proliferation after Ndufa4 KO and overexpression. Neuronal proliferation was assessed using MTS assay. (PNG 35 kb)

High resolution image (TIF 7867 kb)

Supplemental Figure S4 Droplet digital polymerase chain reaction was used to evaluate the microRNA expression in Figures 4B，4C, 5A, and 7C. (A) Droplet digital polymerase chain reaction was used to evaluate the microRNA expression in Figure 4B. (B) Droplet digital polymerase chain reaction was used to evaluate the microRNA expression in Figure 4C. (C) Droplet digital polymerase chain reaction was used to evaluate the microRNA expression in Figure 5A. (D) Droplet digital polymerase chain reaction was used to evaluate the microRNA expression in Figure 7C. **P < 0.01, ***P < 0.001 and ****P < 0.0001. (PNG 314 kb)

High resolution image (TIF 2554 kb)

## Data Availability

The original contributions presented in the study are included in the article/supplementary material and further inquiries can be directed to the corresponding author.

## Abbreviations

*DWM*: Dandy–Walker malformation
*Ndufa4*: NADH dehydrogenase 1 alpha subcomplex 4
*COX*: Cytochrome c oxidase
*DJ1*: Parkinson disease protein 7
*array-CGH*: Array-based comparative genomic hybridization
*CNVs*: Copy number variations
*αMEM*: Minimal Essential Medium—alpha modification
*3′UTRs*: 3′ Untranslated regions
*Homer1*: Human homer protein homolog 1
*Ccnd2*: Cyclin D2
*AD*: Alzheimer’s disease
*BCL-2*: B-cell lymphoma-2
*Cyt C*: Cytochrome C
*RA*: Retinoid acid
*shRNA*: Short hairpin RNA
*PBS*: Phosphate-buffered saline
*KO*: Knockout
*WT*: Wild-type
*DW*: Distilled water
*TEM*: Transmission electron microscopy
*mRNA*: Messenger RNA
*PAGE*: Polyacrylamide gel electrophoresis
*RT-PCR*: Reverse transcription polymerase chain reaction
*cDNA*: Complementary DNA
*mRNA*: Messenger RNA
*ssDNA*: Single-stranded DNA
*FC*: Fold-change
*GAPDH*: Glyceraldehyde 3-phosphate dehydrogenase
*SDS-PAGE*: Sodium dodecyl sulfate–polyacrylamide gel electrophoresis
*PVDF*: Polyvinylidene difluoride
*Tween*: Polyoxyethylene sorbitan fatty acid esters
*SD*: Standard deviation
*Smad3*: Sma- and Mad-related protein 3
*Col4a1*: Collagen 4a1
*FGF17*: Fibroblast growth factor 17
*NUAK1*: NUAK Family SNF1-like Kinase 1
*OD490*: Optical density at 490 nm
*NC*: Negative control
*MTS*: 3-(4,5-Dimethylthiazol-2-yl)-5-(3-carboxymethoxyphenyl)-2-(4-sulfophenyl)-2H-tetrazolium
*7-AAD*: 7-Aminoactinomycin D
*PCR*: Polymerase chain reaction
*H&E*: Hematoxylin and eosin
*TUNEL*: Terminal deoxynucleotidyl transferase-mediated dUTP-biotin nick end labeling assay
*Bax*: B-cell lymphoma-associated X protein
*Bcl-XL*: B-cell lymphoma-extra-large
*RPL7*: Ribosomal Protein L7
