# Supplementary figures and images for: Ndufa4 Regulates the Proliferation and Apoptosis of Neurons via miR-145a-5p/Homer1/Ccnd2
Source: Mol Neurobiol. 2023 Feb 10;60(6):2986–3003. doi: 10.1007/s12035-023-03239-5 (PMC10122635; doi:10.1007/s12035-023-03239-5)

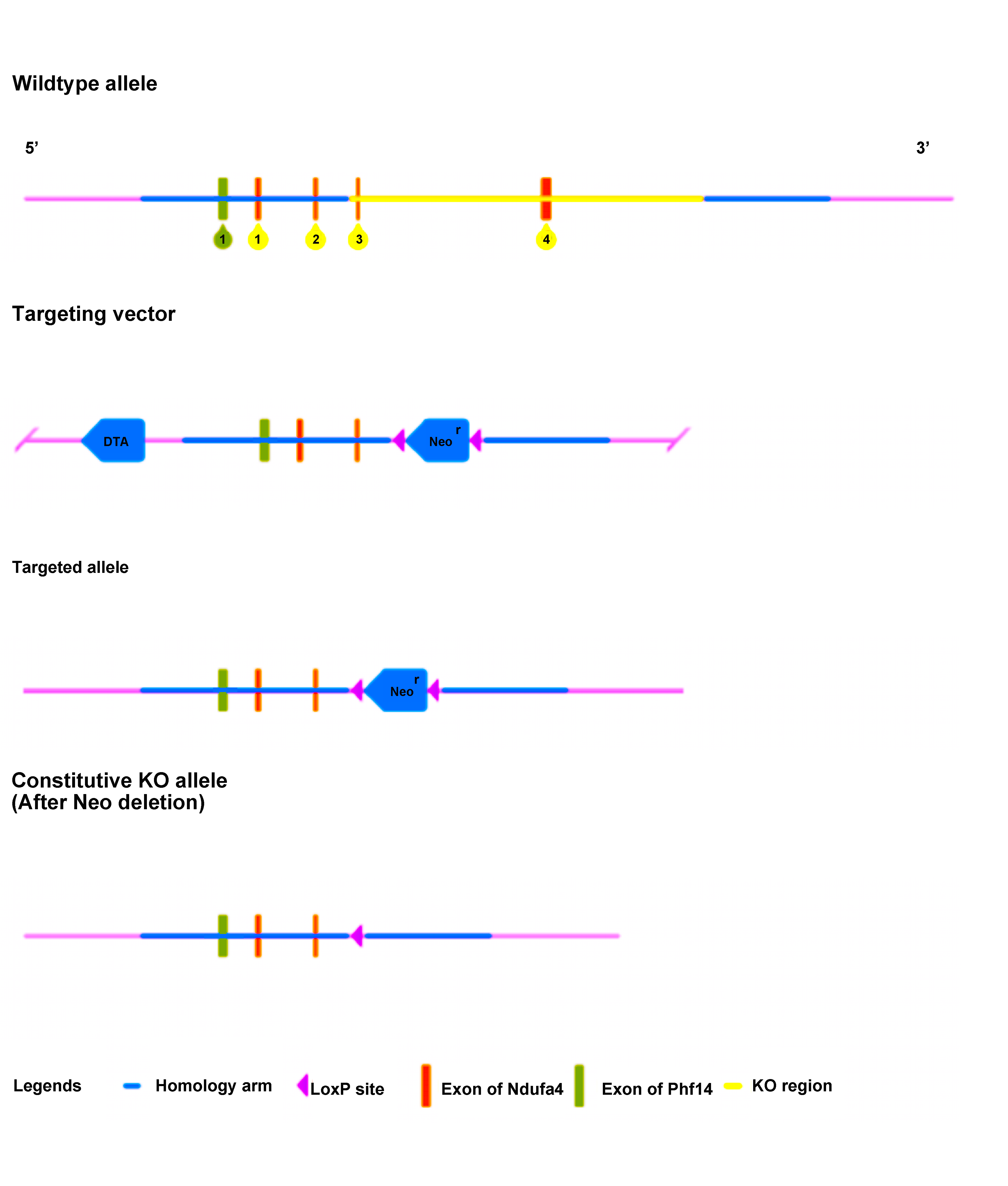

Supplement: Supplementary file 1 — The flow chart of generation of Ndufa4-KO mice. (PNG 1169 kb) [file 12035_2023_3239_Fig8_ESM.png]

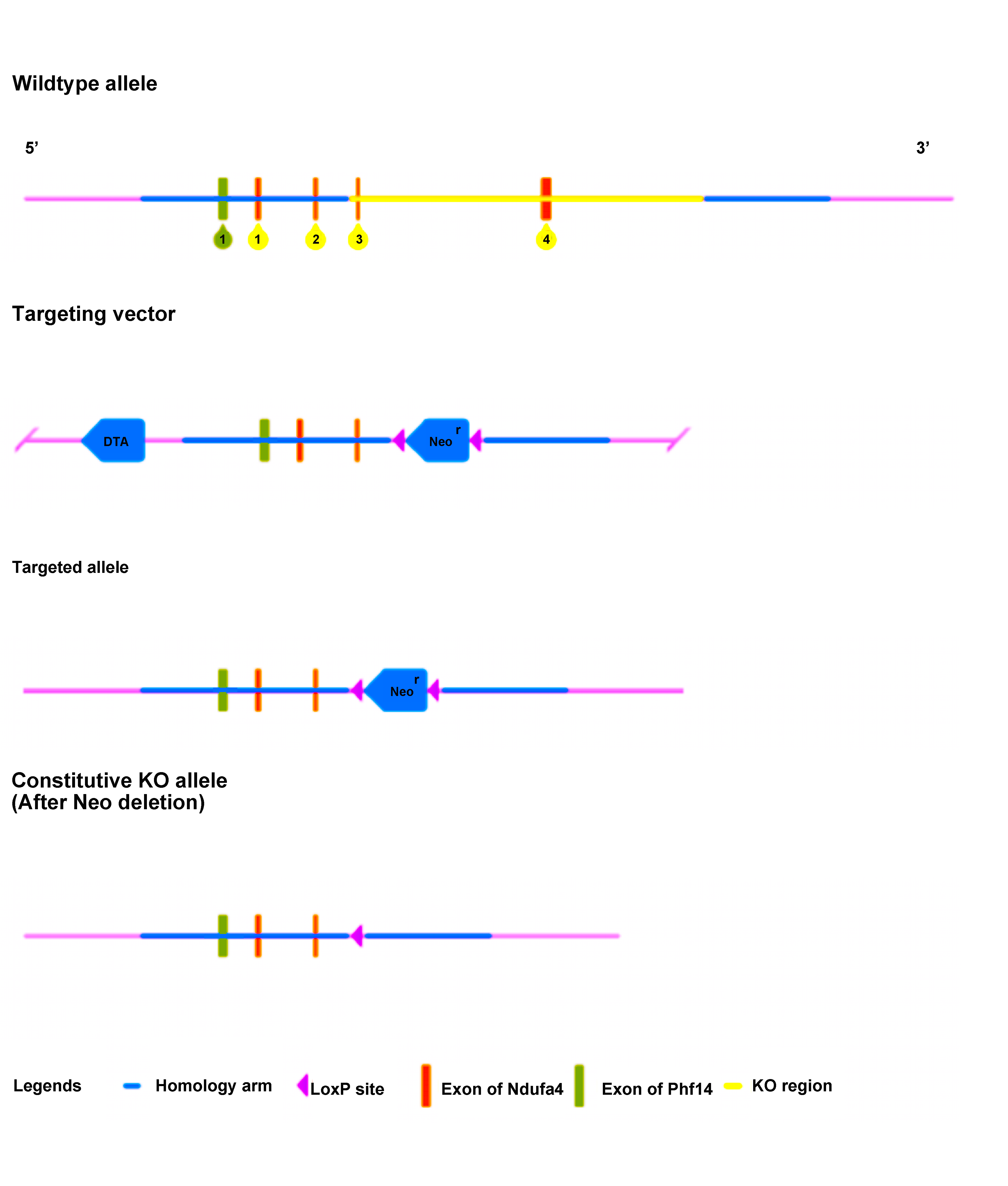

Supplement: Supplementary file 2 — High resolution image (TIF 8219 kb) [file 12035_2023_3239_MOESM1_ESM.tif]

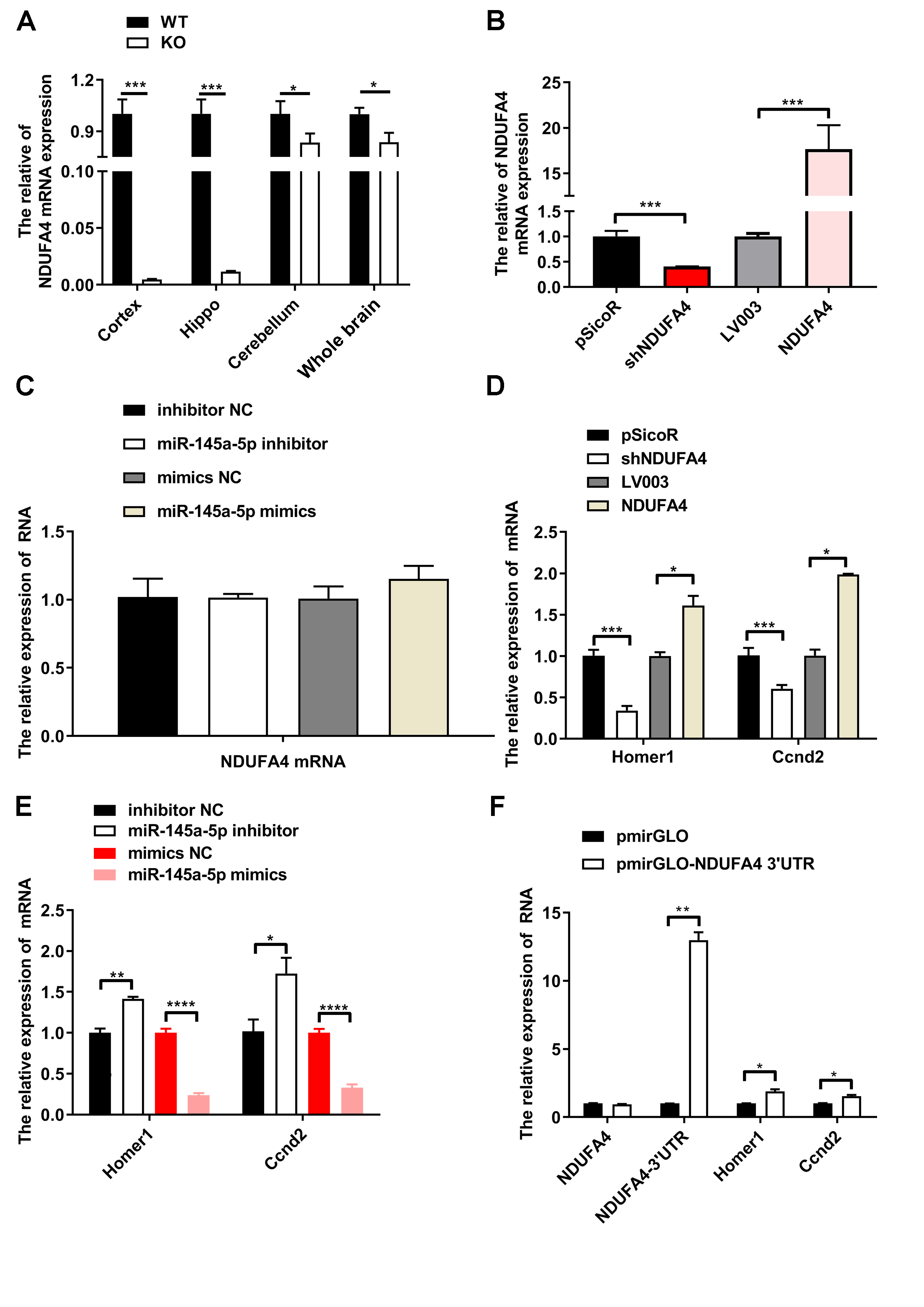

Supplement: Supplementary file 3 — Real time-quantitative polymerase chain reaction results of Figure 1A, 3A, 5A, 6E, 7A, and 7C using RPL7 as reference. (A) Real time-quantitative polymerase chain reaction results of Figure 1A using RPL7 as reference. (B) Real time-quantitative polymerase chain reaction results of Figure 3A using RPL7 as reference. (C) Real time-quantitative polymerase chain reaction results of Figure 5A using RPL7 as reference. (D) Real time-quantitative polymerase chain reaction results of Figure 6E using RPL7 as reference. (E) Real time-quantitative polymerase chain reaction results of Figure 7A using RPL7 as reference. (F) Real time-quantitative polymerase chain reaction results of Figure 7C using RPL7 as reference. *P < 0.05, **P < 0.01, ***P < 0.001 and ****P < 0.0001. RPL7, ribosomal Protein L7. (PNG 476 kb) [file 12035_2023_3239_Fig9_ESM.png]

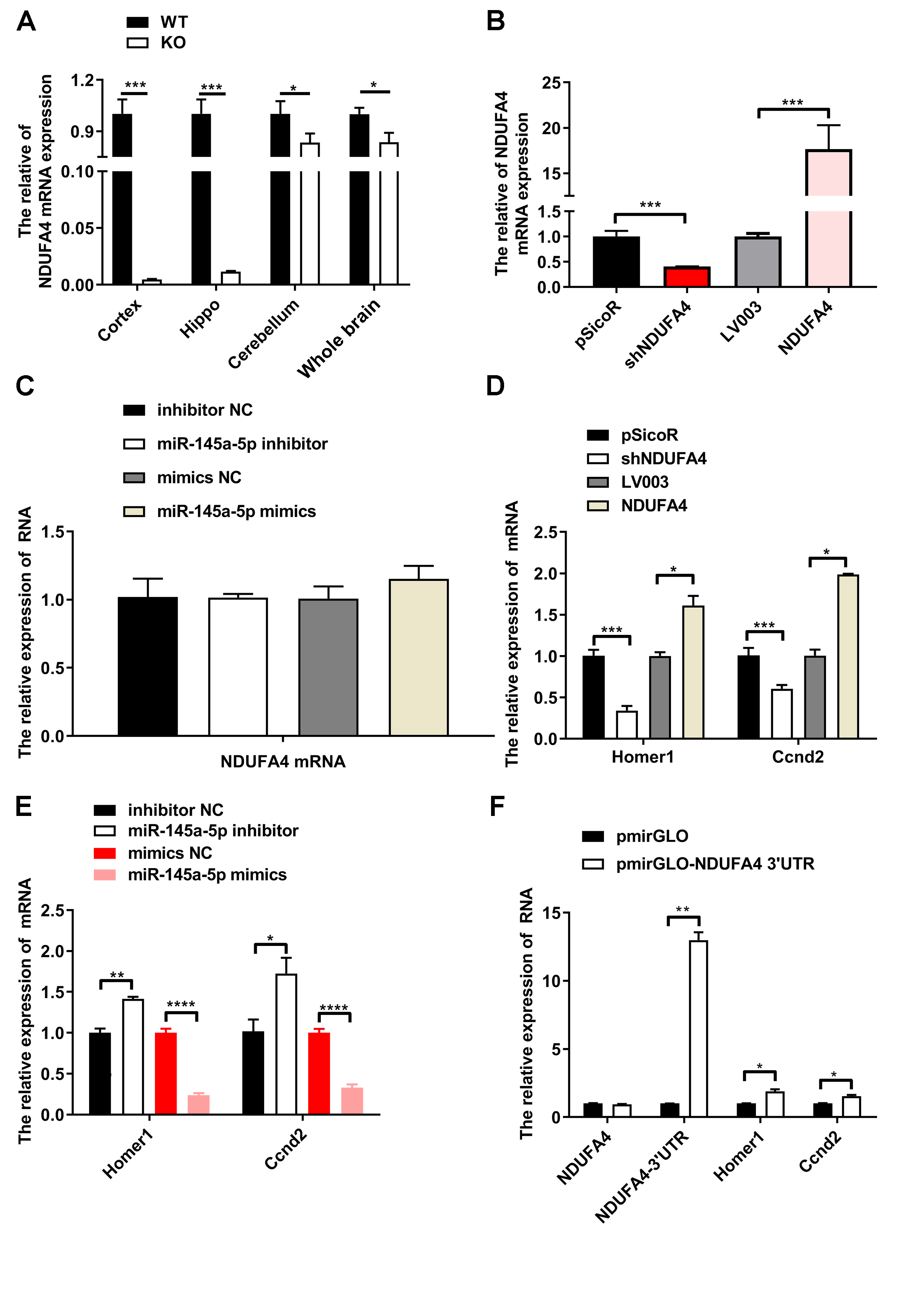

Supplement: Supplementary file 4 — High resolution image (TIF 3083 kb) [file 12035_2023_3239_MOESM2_ESM.tif]

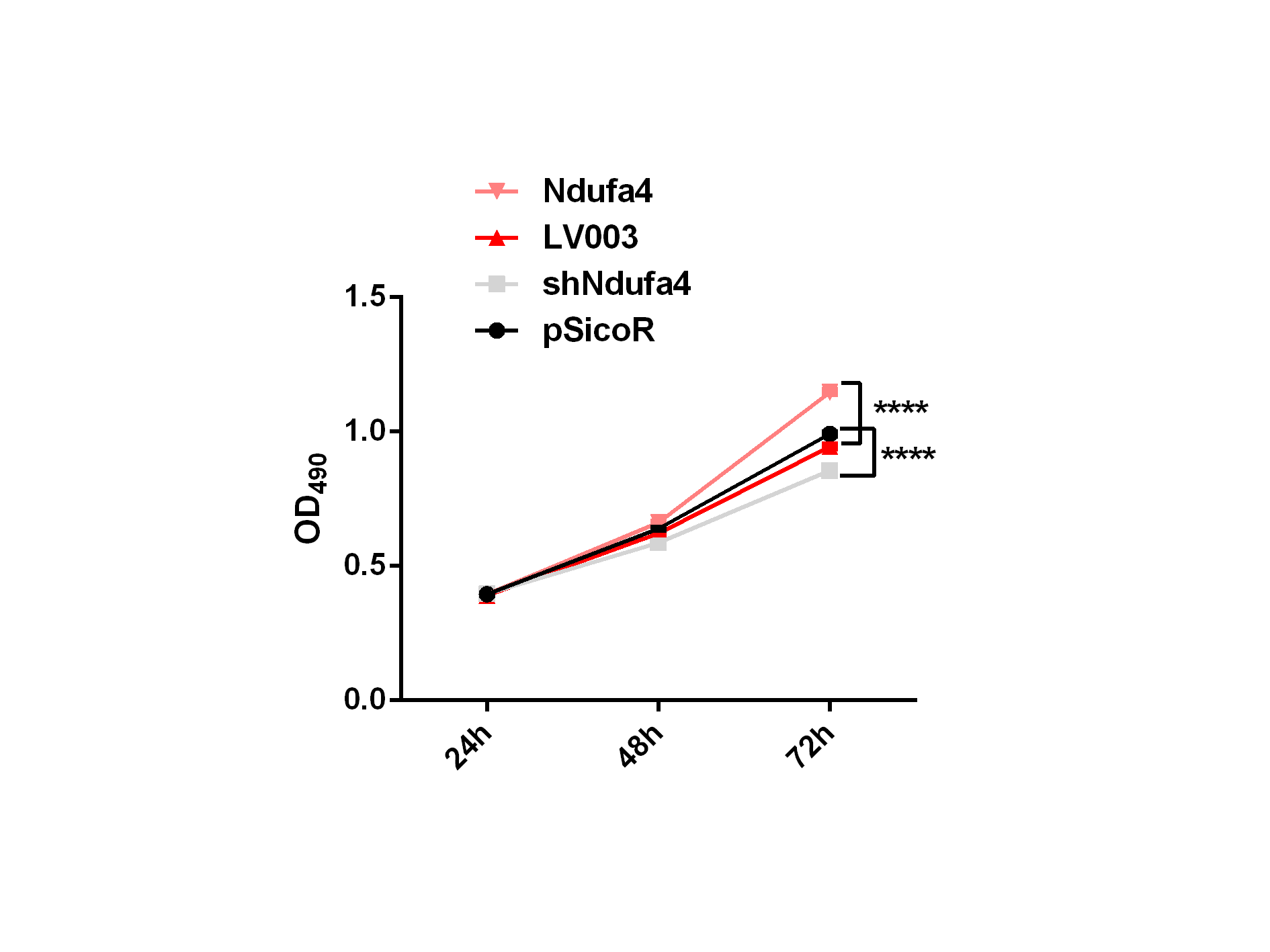

Supplement: Supplementary file 5 — Alterations of NE-4C neural stem cells proliferation after Ndufa4 KO and overexpression. Neuronal proliferation was assessed using MTS assay. (PNG 35 kb) [file 12035_2023_3239_Fig10_ESM.png]

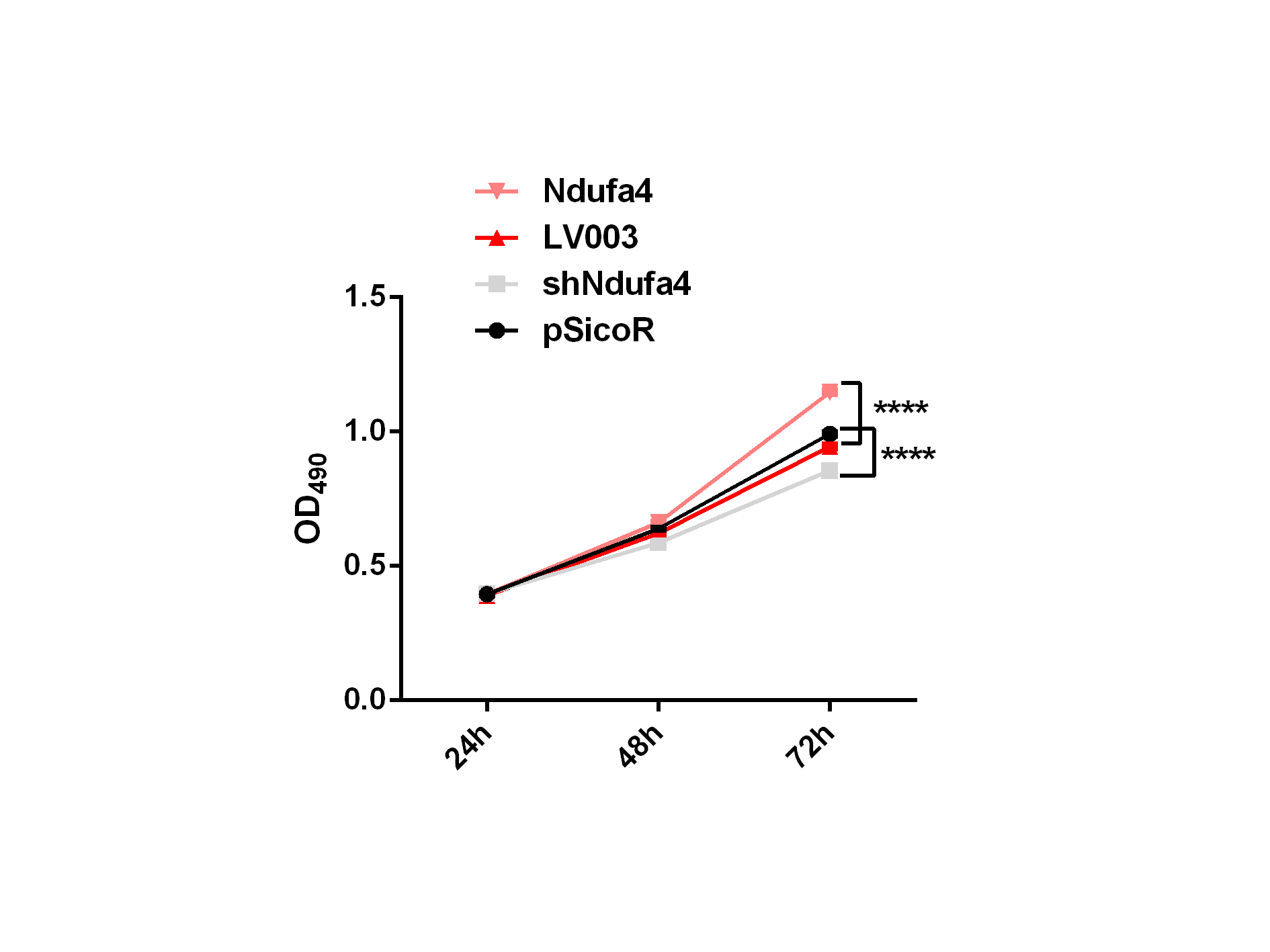

Supplement: Supplementary file 6 — High resolution image (TIF 7867 kb) [file 12035_2023_3239_MOESM3_ESM.tif]

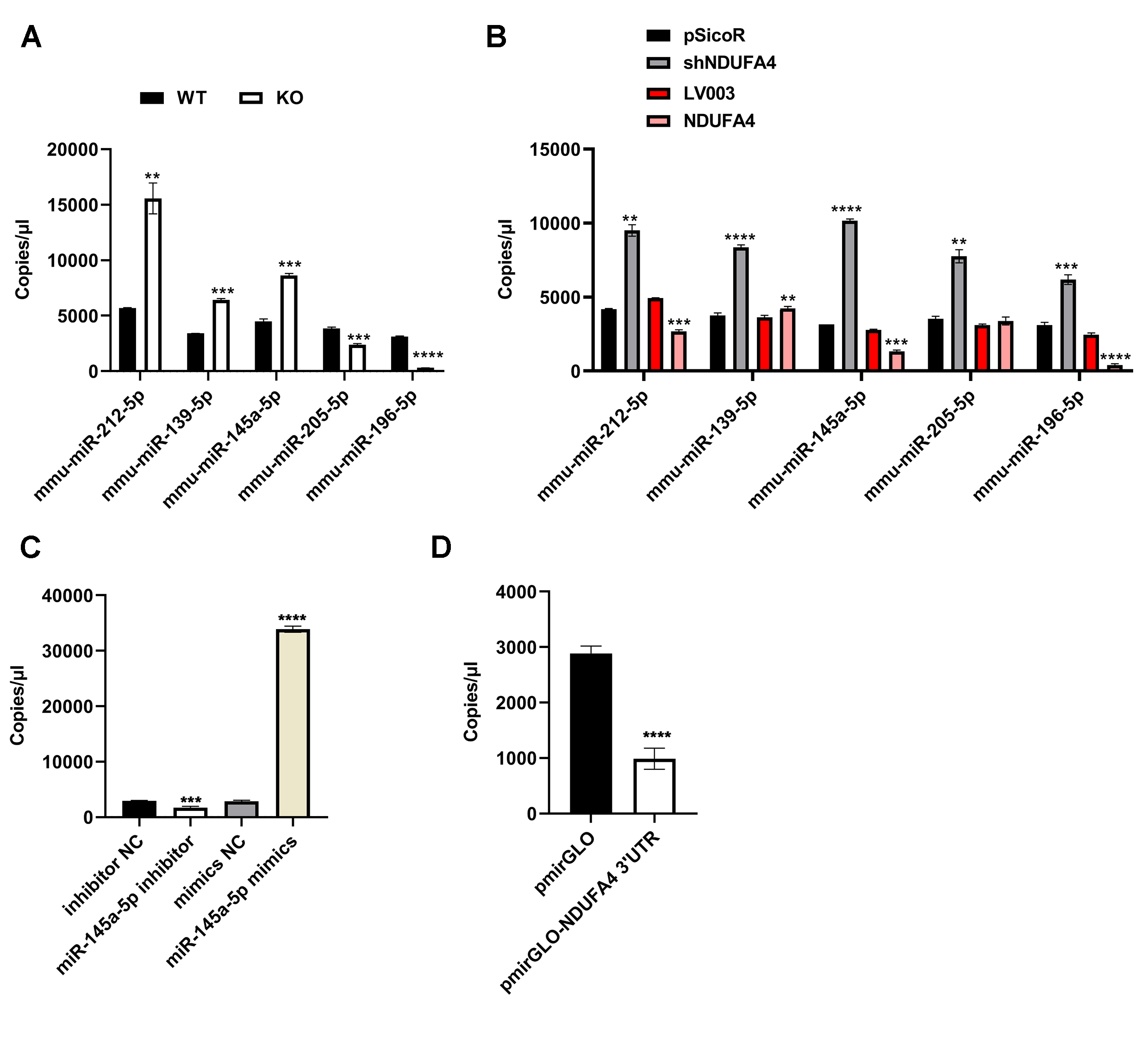

Supplement: Supplementary file 7 — Droplet digital polymerase chain reaction was used to evaluate the microRNA expression in Figures 4B，4C, 5A, and 7C. (A) Droplet digital polymerase chain reaction was used to evaluate the microRNA expression in Figure 4B. (B) Droplet digital polymerase chain reaction was used to evaluate the microRNA expression in Figure 4C. (C) Droplet digital polymerase chain reaction was used to evaluate the microRNA expression in Figure 5A. (D) Droplet digital polymerase chain reaction was used to evaluate the microRNA expression in Figure 7C. **P < 0.01, ***P < 0.001 and ****P < 0.0001. (PNG 314 kb) [file 12035_2023_3239_Fig11_ESM.png]

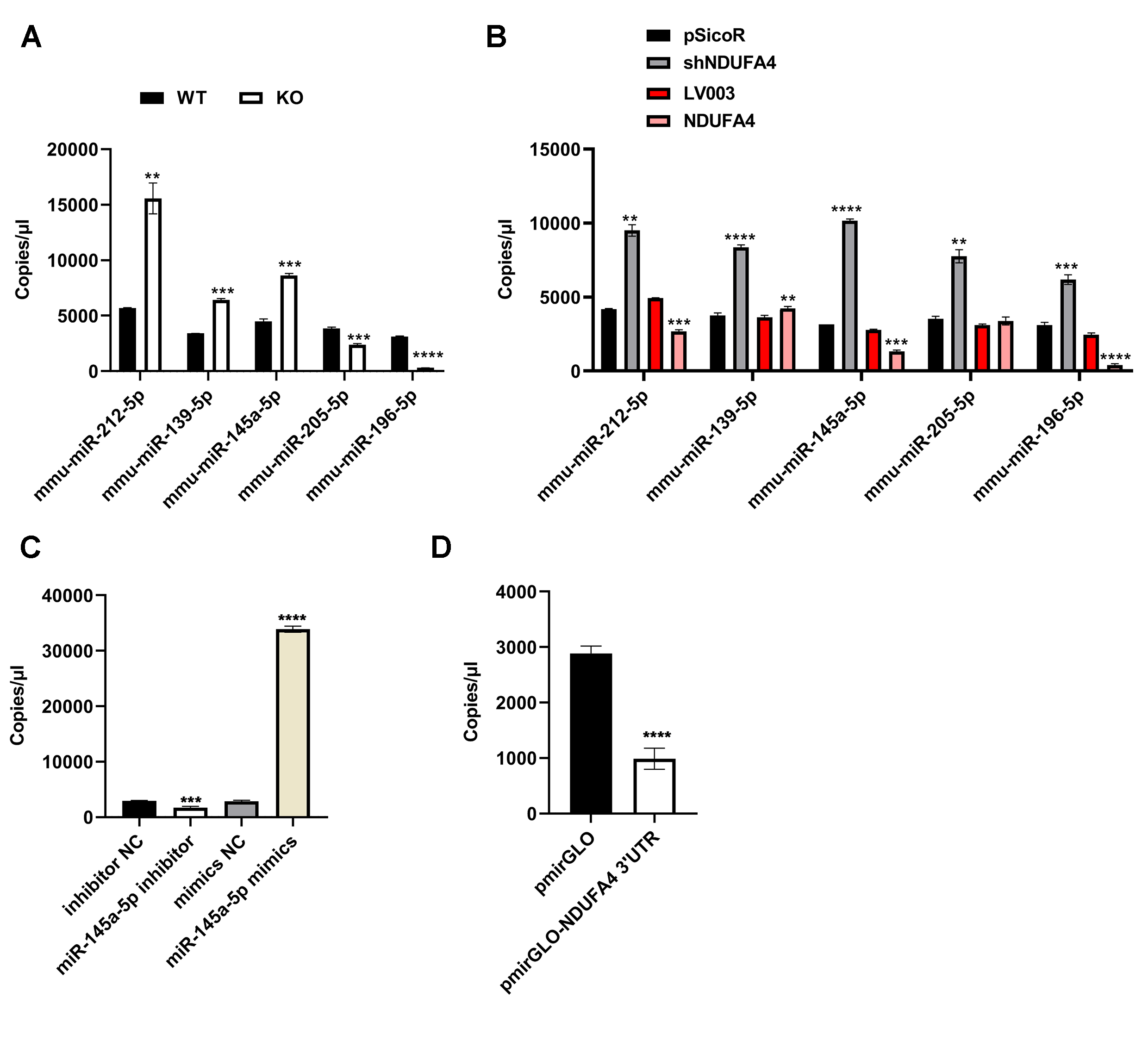

Supplement: Supplementary file 8 — High resolution image (TIF 2554 kb) [file 12035_2023_3239_MOESM4_ESM.tif]
